# Dietary Nucleotides Improve Growth of Juvenile *Eriocheir sinensis* Under a Low‐Fish Meal Diet via Enhanced Feed Intake and Gut Health

**DOI:** 10.1155/anu/8633112

**Published:** 2025-12-09

**Authors:** Song Wang, Wen Li, Yuxi Yan, Erchao Li, Xiaodan Wang, Jian G. Qin, Liqiao Chen

**Affiliations:** ^1^ School of Life Sciences, East China Normal University, Shanghai, 200241, China, ecnu.edu.cn; ^2^ College of Science and Engineering, Flinders University, Adelaide, 5001, South Australia, Australia, flinders.edu.au

**Keywords:** feed intake, growth performance, gut microbiota, low-fish meal diet, nucleotides

## Abstract

Fish meal replacement in aquaculture feeds requires supplementing functional additives to maintain animal performance. Nucleotides are a promising supplement in finfish, but their role in crustaceans remains poorly understood, particularly under challenging low‐fish meal dietary conditions. This study evaluated dietary nucleotide supplementation effects on juvenile Chinese mitten crabs (*Eriocheir sinensis*) fed low‐fish meal diets. Juvenile crabs (0.58 ± 0.01 g, first‐year cohort) were distributed across 35 tanks with 1400 individuals total, creating seven dietary treatments (*n* = 5 replicates, 40 crabs per replicate). Over 56 days, crabs received either a control diet (35% fish meal) or six low‐fish meal diets (15% fish meal) supplemented with graded nucleotide concentrations (0, 0.3, 0.6, 0.9, 1.2, or 2.4 g/kg). Nucleotide supplementation increased feed intake in a dose‐dependent manner (*p* < 0.05); at 0.9 g/kg, intake was restored to the control level (*p* > 0.05). This higher intake coincided with increased digestive enzyme activities (α‐amylase (α‐Ams), trypsin (Try), and lipase) and improved growth (weight gain and specific growth rate; *p* < 0.05), with 0.9 g/kg or higher restoring growth to levels not significantly different from the high‐fish meal control. Mechanistically, supplementation restored intestinal health by preserving morphology and lowering inflammation‐related gene expression. Furthermore, it bolstered hepatopancreatic antioxidant defenses (a key transcriptomic finding) and favorably restructured the gut microbiota, which correlated with host performance (*p* = 0.007). Thus, nucleotide supplementation mitigates the adverse effects of low‐fish meal diets by improving feed intake and intestinal health. The optimal level for growth was 0.85 g/kg based on broken‐line regression.

## 1. Introduction

Aquaculture’s reliance on formulated aquafeeds [1] has been challenged by the static production and sustainability concerns of fish meal [2, 3], which is traditionally preferred for its high palatability and balanced nutrition [4, 5]. This supply‐demand gap necessitates a shift toward sustainable alternatives, such as widely available plant‐derived proteins (PPs) [6]. However, high inclusion of PPs can compromise the performance of aquatic species, especially carnivorous crustaceans [7], through two primary mechanisms. First, PPs possess low palatability [8, 9], which curtails feed consumption and limits nutrient acquisition [10]. Second, PPs often contain antinutritional compounds [11] and nutritional imbalances like phytic acid and amino acid deficiencies [12]. Low feed intake and these nutritional deficiencies, including essential amino acid imbalances and the presence of antinutritional factors, are often associated with slow growth, low feed utilization efficiency, and poor intestinal integrity. These interconnected problems markedly limit the replacement levels of fish meal in commercial feeds [13].

Addressing the constraint of reduced palatability and nutritional imbalance in plant protein sources requires using functional feed additives. Among these, nucleotides are recognized as a valuable substance due to their diverse biological functions such as energy transfer, metabolism, and cell signaling [14]. Replacing fish meal with plant proteins significantly reduces the natural dietary supply of these compounds, which are abundant in fish meal but scarce in plant ingredients [15, 16]. Consequently, under some conditions, such as low‐fish meal feeding, nucleotides may become conditionally essential and require supplementation. A key aspect of their benefit is related to palatability. Specific nucleotides, particularly inosine monophosphate (IMP) and guanosine monophosphate (GMP), act as potent umami substances and key palatability enhancers in aquatic feeds [17, 18]. These compounds are known to stimulate feeding behavior via a gustatory mechanism in various fish species [19], suggesting their potential to improve feed acceptance when formulating a diet with low fish meal.

Beyond palatability enhancement, nucleotides have broader physiological influences. For instance, they can improve digestibility coefficients by increasing digestive enzyme secretion [20]. They may also enhance intestinal epithelial development through accelerated enterocyte maturation and increased villus height [21]. However, the physiological benefits vary with species and the baseline diet composition. Significant knowledge gaps exist regarding nucleotide applications in crustaceans, particularly under a challenging low‐fish meal dietary condition. The efficacy of nucleotide supplementation exhibits species‐specific variability [22, 23]. Furthermore, recent evidence reveals response variations dependent on baseline fish meal levels. While nucleotide supplementation significantly improves growth performance, it often provides no benefits when fish meal levels are already high [24]. This suggests that nucleotides are a limiting nutrient, specifically in plant‐based formulation, where the natural nucleotide content is low in the dietary ingredients.

The Chinese mitten crab (*Eriocheir sinensis*), an economically significant decapod in Asian aquaculture, represents an ideal model organism for investigating nucleotide‐diet interactions. This suitability stems from its documented sensitivity to dietary fish meal replacement [25, 26] and its digestive physiology, where the hepatopancreas serves as the central organ for nutrient metabolism [27]. The current investigation tests the hypothesis that dietary nucleotide supplementation can enhance feed palatability and stimulate feed intake in juvenile *E. sinensis* fed low‐fish meal diets. This study addresses the poor feed acceptance and low feed consumption associated with a plant protein‐based formulation. It was hypothesized that the multifunctional properties of nucleotides would mitigate other adverse physiological effects of low‐fish meal diets by improving digestive capacity, maintaining intestinal integrity, and favorably modulating the gut microbiota. To elucidate these functional mechanisms, the experimental approach incorporated comprehensive physiological assessments, including targeted "omics" analyses of the hepatopancreas and gut. Specifically, hepatopancreatic transcriptomics was employed to identify the direct molecular pathways (e.g., metabolic and antioxidant) regulated by nucleotide supplementation itself within the low‐fish meal context. Concurrently, gut microbiota analysis was utilized to assess a potential “rescue” effect—evaluating whether nucleotides could restore a microbiota community disrupted by the low‐fish meal diet relative to a high‐fish meal healthy baseline.

## 2. Materials and Methods

### 2.1. Experimental Diets

The formulation and nutrient content of the seven isonitrogenous and isolipidic experimental diets are outlined in Table 1. Protein in these diets was primarily supplied by soybean meal, cottonseed meal, and fish meal. Lipids were sourced from fish oil, soybean oil, soy lecithin, and cholesterol, with pregelatinized starch serving as the carbohydrate. One diet, designated as the control (Con), had a fish meal content of 35%. The subsequent six diets featured a reduced fish meal level (15%) and incorporated a nucleotide mixture at varying inclusion levels: 0, 0.3, 0.6, 0.9, 1.2, or 2.4 g/kg.

**Table 1:** Composition and source details of dietary supplements (g/kg dry matter).

Ingredients	Con	0	0.3	0.6	0.9	1.2	2.4
Fish meal	350	150	150	150	150	150	150
Soybean meal	112	240	240	240	240	240	240
Cottonseed meal	112	240	240	240	240	240	240
Fish oil	14.5	20	20	20	20	20	20
Soybean oil	14.5	20	20	20	20	20	20
Lecithin	10	10	10	10	10	10	10
Cholesterol	5	5	5	5	5	5	5
Choline chloride	5	5	5	5	5	5	5
Nucleotides^a^	0	0	0.3	0.6	0.9	1.2	2.4
Butylated hydroxytoluene	1	1	1	1	1	1	1
Pregelatinized starch	180	180	180	180	180	180	180
Vitamin premix^b^	40	40	40	40	40	40	40
Mineral premix^c^	20	20	20	20	20	20	20
Alginate	20	20	20	20	20	20	20
Monocalcium phosphate	10	10	10	10	10	10	10
Coated lysine^d^	0	7.8	7.8	7.8	7.8	7.8	7.8
Coated methionine^e^	0	5.4	5.4	5.4	5.4	5.4	5.4
Cellulose	106	25.8	25.5	25.2	24.9	24.6	23.4
Proximate composition (%)							
Crude protein	39.26	39.29	38.95	39.39	38.67	38.58	39.21
Crude lipid	7.85	7.69	7.78	7.45	7.77	7.84	7.82

Abbreviations: AMP, adenosine monophosphate; CMP, cytidine monophosphate; GMP, guanosine monophosphate disodium; IMP, inosine monophosphate disodium; UMP, uridine monophosphate disodium.

^a^The nucleotide mixture was supplied by Nanjing Tonkai Biological Technology Co., Ltd. (Nanjing, China). It consisted of purified AMP, GMP, IMP, UMP, and CMP mixed in an equal ratio (1:1:1:1:1 by weight).

^b^Each 100 g of the vitamin premix contained: thiamin hydrochloride (0.15 g), retinol acetate (0.043 g), cholecalciferol (0.0075 g), riboflavin (0.0625 g), folic acid (0.025 g), ca. pantothenate (0.3 g), pyridoxine hydrochloride (0.225 g), niacin (0.3 g), α‐tocopherol acetate (0.5 g), ascorbic acid (0.5 g), menadione (0.05 g), biotin (0.005 g), paraaminobenzoic acid (0.1 g), and inositol (1 g). The final volume was adjusted to 100 g using α‐cellulose as a carrier.

^c^Each 100 g of the mineral premix contained: KH_2_PO_4_ (21.5 g), CuCl_2_·2H_2_O (0.015 g), KCl (2.8 g), AlCl_3_·6H_2_O (0.024 g), MnSO_4_ · 6H_2_O (0.143 g), CoCl_2_ · 6H_2_O (0.14 g), Ca(H_2_PO_4_)_2_ (26.5 g), ZnSO_4_ · 7H_2_O (0.476 g), MgSO_4_ · 7H_2_O (10.0 g), CaCO_3_ (10.5 g), Fe‐citrate (1 g), NaH_2_PO_4_ (10.0 g), KI (0.023 g), and calcium lactate (16.50 g). The mixture was standardized to 100 g with α‐cellulose.

^d,e^Coated lysine and coated methionine were supplied by King Techina Group (Hangzhou, China). Both coated products had an amino acid purity of 50%, with the remaining 50% dextrin.

### 2.2. Experimental Crabs

The study was conducted under the ethical guidelines and approval (Number f20190101) of the Experimental Animal Administration Committee of East China Normal University. The ARRIVE 2.0 checklist for this study is provided as Supporting Information 2: File S2. Juvenile Chinese mitten crabs (*E. sinensis*; 0.58 ± 0.01 g) were sourced from a commercial farm in Chongming, Shanghai, and acclimated to laboratory conditions for 2 weeks. Subsequently, 1400 healthy crabs (with intact limbs) were distributed randomly into 35 aerated 300‐L tanks (100 cm × 50 cm × 60 cm), creating five replicate tanks per treatment, each stocked with 40 individuals. Tank positions within the room were randomized to minimize possible location effects (e.g., light and water inflow); replicates of the same treatment were interspersed so that no two replicates were adjacent. All tanks shared the same water source and aeration regime. Handling order for measurements was balanced across treatments. Shelters, consisting of one six‐layer net (30 cm × 30 cm) and two sets of five tied‐plastic tubes per tank, were provided to reduce aggressive interactions. Throughout the 8‐week experimental period, crabs received feed to apparent satiation twice daily (08:30 and 16:30), with molting events recorded on a daily basis. Optimal water quality was sustained by a daily one‐third water exchange, ensuring temperature was kept at 25 ± 0.5°C, pH at 7.4–7.8, dissolved oxygen at > 7.0 mg/L, and ammonia nitrogen at <0.05 mg/L.

### 2.3. Sample Collection

At the end of the 56‐day trial, crabs were fasted for 24 h and final body weights were recorded. Daily feed intake was tracked by collecting and weighing uneaten feed after each meal. During the final 7 days, 1‐h feed intake (1 h FI) was measured as 1 h FI (g/g crab) = [*W*_f_ – *W*_u_(1 + *i*)]/*W*_T_, where *i* = 0.08 is the leaching rate for this feed, *W*_f_ is the offered feed, *W*_u_ is the recovered uneaten feed, and *W*_T_ is the tank biomass at measurement [28]. Mortalities were removed and logged daily and feed conversion ratio (FCR) was calculated from feed consumption and biomass gain, including the weight gain of dead crabs. Protein retention (PR) used Day 0 whole‐body protein as the initial value and Day 56 as the final value. Five crabs per tank were collected for whole‐body composition and stored at −20°C. Remaining crabs were euthanized on ice for tissue collection. Brain and thoracic ganglia, hepatopancreas, and intestine were dissected, flash‐frozen in liquid nitrogen, and stored at −80°C. Three hepatopancreas samples per treatment were used for RNA‐seq, and five intestinal samples per treatment for microbiota analysis.

### 2.4. Analysis Methods

#### 2.4.1. Proximate Composition and Diet Nucleotide Analysis

Standard AOAC [29] procedures were employed for the proximate analysis of both experimental diets and whole‐body crab samples. For moisture determination, diet samples were oven‐dried to a constant weight at 105°C, whereas whole‐body samples underwent lyophilization for 72 h using a Christ Beta 2–4 LD plus LT freeze‐dryer (Germany). Prior to analysis, all dried materials were thoroughly homogenized with a laboratory mill. The Kjeldahl method (Kjeltec 8200, Foss, Sweden) was utilized to quantify crude protein content (*N* × 6.25). Crude lipid levels were ascertained through Soxhlet extraction (1000 mL, Soxhlet apparatus). Ash content was measured by incinerating samples in a muffle furnace (PCD‐E3000 Series, Japan) at 550°C for 8 h, subsequent to initial carbonization. To determine the concentrations of specific nucleotides (CMP, AMP, UMP, GMP, and IMP) within all formulated diets, high‐performance liquid chromatography was performed in accordance with the Chinese national standard GB 5413.40‐2016. The analytical outcomes for each diet are provided in Supporting Information 1: Table S1.

#### 2.4.2. Digestive and Absorptive Enzyme Activities

To assess digestive and absorptive enzyme activities, hepatopancreas and intestinal tissues were first homogenized (at a 1:9 weight/volume ratio) in ice‐cold physiological saline (0.85% NaCl solution). The homogenates were then subjected to centrifugation at 1500 × *g* for 15 min at 4°C. The resulting supernatants were carefully collected and kept at −80°C pending analysis. The activities of *α*‐amylase (*α*‐Ams), trypsin (Try), lipase, and *γ*‐glutamyl transferase (*γ*‐GT) were quantified using commercially available assay kits sourced from the Nanjing Jiancheng Bioengineering Institute (China), strictly adhering to the manufacturer’s specified protocols.

#### 2.4.3. Gene Expression Analysis

The procedures for RNA extraction and subsequent RT‐qPCR analysis adhered to the methodology previously detailed by Li et al. [28]. For the isolation of total RNA, RNAiso Plus reagent (TaKaRa, Japan) was utilized. Prior to cDNA synthesis, the quality (ensuring a 260/280 nm absorbance ratio between 1.8 and 2.0) and integrity of the extracted RNA were confirmed. First‐strand cDNA was synthesized using the HiScript III RT SuperMix (Vazyme Biotech, China). Real‐time quantitative PCR reactions were then carried out on a CFX96 system (Bio‐Rad, USA) employing ChamQ Universal SYBR qPCR Master Mix. All primer sequences used are listed in Supporting Information 1: Table S2. Gene expression levels were quantified relative to the reference gene *s27*, using the 2^−*ΔΔ*Ct^ calculation method.

#### 2.4.4. Histological Analysis

For histological examination, intestinal samples were promptly fixed in Bouin’s solution upon collection. The fixed tissues subsequently underwent a standard processing protocol, which included sequential dehydration in a graded ethanol series, clearing with xylene, followed by infiltration and embedding in molten paraffin wax. Paraffin‐embedded tissue blocks were then sectioned, and the resulting sections were mounted onto glass slides and allowed to dry. These sections were subsequently stained with hematoxylin and eosin (HE). Finally, the stained slides were examined under a light microscope (Nikon, Japan), and representative images were captured for morphological evaluation.

#### 2.4.5. Hepatopancreatic Transcriptomic Analyses

Hepatopancreas samples from the 0 and 0.9 g/kg nucleotide groups (15% fish meal diets; *n* = 3) were processed for transcriptomic analysis. Following RNA extraction (Section 2.4.3), libraries were prepared using Illumina Stranded mRNA Prep kits and sequenced on an Illumina NovaSeq X Plus platform (2 × 150 bp paired‐end; Shanghai Majorbio Bio‐Pharm Biotechnology Co., Ltd.). HISAT2 software was employed to align the quality‐filtered clean reads against the *E. sinensis* reference genome (GCF_024679095.1). Gene expression was quantified as fragments per kilobase (FPKM) of transcript per Million mapped reads. Identification of differentially expressed genes (DEGs) was performed with DESeq2, applying thresholds of |log_2_ fold change| > 2 and an adjusted *p*‐value < 0.05. Functional analysis included Kyoto Encyclopedia of Genes and Genomes (KEGG) pathway enrichment (adjusted *p*‐value < 0.05) and protein–protein interaction network construction (STRING v12.0, visualized with Cytoscape).

#### 2.4.6. Gut Microbiota Analysis

Intestinal microbiota was analyzed from three representative groups: control (35% fish meal), 15% fish meal with 0 g/kg nucleotide, and 15% fish meal with 0.9 g/kg nucleotide (*n* = 5 per group). These three treatments represented a standard control, without nucleotide supplementation in low‐fish meal diets, and optimal supplementation in low‐fish meal diets. Genomic DNA was extracted using the E.Z.N.A. Soil DNA Kit (Omega Biotek, USA) and quality verified before processing. The V3–V4 regions of the 16S rRNA gene were amplified using primers 338F and 806R. PCR was performed in triplicate, and products were sequenced on an Illumina NovaSeq PE250 platform (Shanghai Majorbio Bio‐pharm Technology Co., Ltd.). Sequence processing included quality filtering (fastp), paired‐end read merging (FLASH), and OTU clustering at 97% similarity (UPARSE algorithm). Taxonomy was assigned using the RDP Classifier against the Silva database (v138) with a confidence threshold of 0.7. Diversity analyses included alpha diversity indices (Shannon, Simpson, ACE, and Chao) and beta diversity assessment through principal coordinate analysis (PCoA) based on Bray–Curtis distances, with significance tested by PERMANOVA (999 permutations). Key microbial taxa were identified through specificity‐occupancy analysis [30]. Co‐occurrence networks were constructed using significant Spearman correlations (|*r*| ≥ 0.5, *p* < 0.05) between prevalent OTUs and visualized using Cytoscape.

### 2.5. Calculations and Statistical Analyses

Growth performance and feed utilization parameters were calculated as follows:

Weight gain WG,%=100×Final weight−initial weight/initial weight,

Specific growth rate SGR,%/day=10056×ln final weight−ln initial weight/ days,

Molting frequency MF,%=1002××Number of molts/initial number+final number of crabs,

Survival %=100×Final number of crabs/initial number of crabs,

Feed conversion ratio FCR=Feed intake/final weight−initial weight+weight gain of dead crabs,

Protein efficiency ratio PER=Final weight−initial weight/protein intake,

Protein retention PR,%=100×Final protein −initial protein content/protein intake,

where initial protein content was determined from the baseline samples collected at Day 0 and the final protein content from samples collected at Day 56.

All analyses used tank means as the experimental unit (*n* = 5 per treatment) to avoid pseudo‐replication. Statistical processing of the data was performed with SPSS software (version 20.0; Chicago, IL, USA). Prior to analysis, datasets were examined for normal distribution using the Shapiro–Wilk test and for variance homogeneity via Levene’s test. To evaluate differences across the six groups receiving 15% fish meal diets, a one‐way analysis of variance (ANOVA) was employed. Significant effects identified by ANOVA (*p* < 0.05) were further explored using Duncan’s multiple range test for post hoc mean comparisons. Data are presented as mean values ± standard error of the mean (SEM). Furthermore, comparisons between the control group (35% fish meal) and each of the six 15% fish meal diet groups were made individually using independent samples *t*‐tests. For all statistical evaluations, a *p*‐value less than 0.05 was adopted as the threshold for statistical significance. To determine if responses exhibited linear or quadratic trends with increasing nucleotide levels, orthogonal polynomial contrasts were applied. Broken‐line regression analysis, following the model described by Robbins et al. [31], was employed to estimate the optimal dietary nucleotide concentration for both WG and 1 h FI. Microbiota data were analyzed using redundancy analysis (RDA) in R (“vegan” package) to relate gut microbiota composition (Hellinger‐transformed OTUs) to host physiological variation. Principal components explaining > 80% variance from standardized host data were used as constraining variables. The significance of the constraints was determined via 999 permutation tests.

## 3. Results

### 3.1. Feed Intake and Appetite‐Related Gene Expression

One‐hour feed intake (1 h FI) was significantly lower in the 0 g/kg nucleotide group compared to the control (*p* < 0.01). Supplementation with 0.6 and 0.9 g/kg nucleotide significantly increased 1 h FI relative to the 0 g/kg group (*p* < 0.05; Figure 1A). At the 0.9 g/kg nucleotide level, 1 h FI was comparable to that of the control group (*p* > 0.05). Broken‐line regression estimated the optimal dietary nucleotide level for 1 h FI as 0.76 g/kg (Figure 1B). Dietary nucleotides also modulated the expression patterns of appetite‐related genes in neural tissues (Figure 1C, D). Nucleotide supplementation tended to upregulate orexigenic signals and downregulate anorexigenic signals (e.g., LEPR and TOR pathway components) relative to the 0 g/kg group. Similar expression modulation patterns occurred in both cranial and thoracic ganglia.

Figure 1Effects of dietary nucleotide supplementation on feed intake and appetite‐related gene expression in juvenile *Eriocheir sinensis* fed low‐fish meal diets. (A) 1‐h feed intake (1 h FI, g/g crab). (B) Broken‐line regression analysis of 1 h FI versus dietary nucleotide concentration. (C, D) Relative mRNA expression of appetite‐related genes in cranial ganglia (C) and thoracic ganglia (D), with gene names listed on the left. Data are presented as means ± SEM (*n* = 5). Different lowercase letters indicate significant differences among 15% fish meal treatments (*p* < 0.05); asterisks indicate significant differences compared to the 35% fish meal control (*p* < 0.05,  ^∗^ *p* < 0.01).

**Figure:**
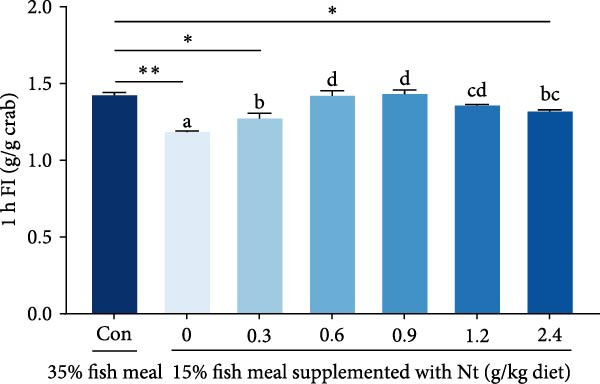
(A)

**Figure:**
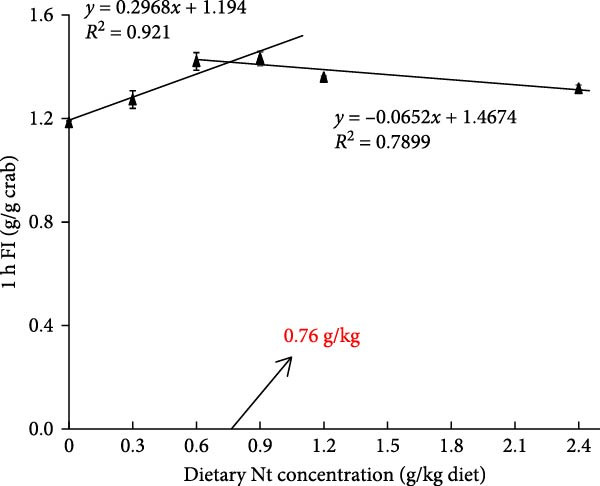
(B)

**Figure:**
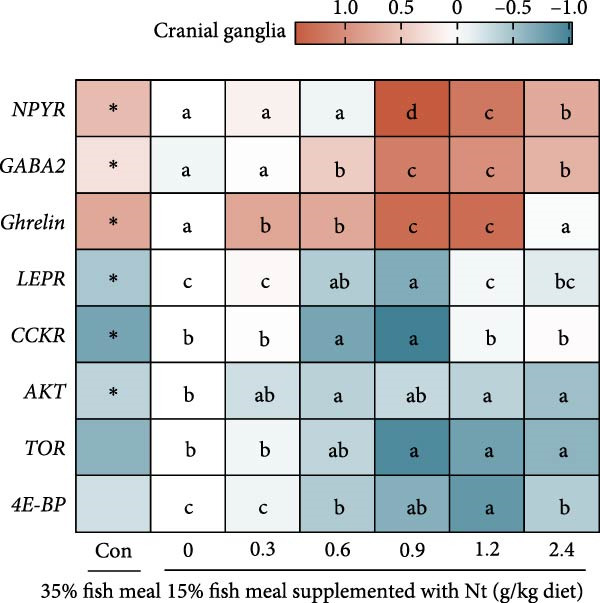
(C)

**Figure:**
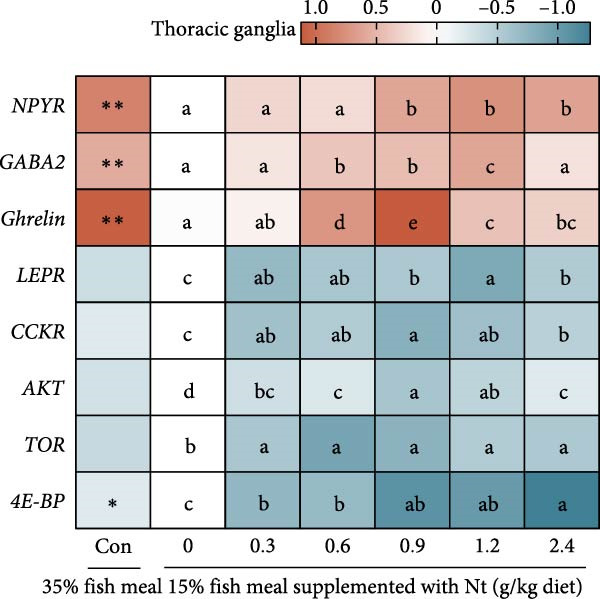
(D)

### 3.2. Digestive Enzyme Activities

Activities of key digestive enzymes in both the hepatopancreas and intestine were generally lower in crabs fed the 15% fish meal diet without nucleotide supplementation (0 g/kg) compared to the control group (Figure 2A–H). Dietary nucleotide supplementation significantly increased these enzyme activities relative to the 0 g/kg group (*p* < 0.05). Notably, supplementation with 0.9 g/kg nucleotide restored the activities of most key digestive enzymes (including *α*‐Ams, Try, and lipase in both tissues) to a level comparable with the control group (*p* > 0.05). An exception was observed for hepatopancreatic *γ*‐GT activity, which significantly exceeded control levels in the 0.9 g/kg nucleotide group (*p* < 0.05; Figure 2G).

Figure 2Effects of dietary nucleotide supplementation on digestive and absorptive enzyme activities in juvenile *Eriocheir sinensis* fed low‐fish meal diets. Activities of *α*‐amylase (*α*‐Ams) in (A) hepatopancreas and (B) intestine; trypsin (Try) in (C) hepatopancreas and (D) intestine; lipase in (E) hepatopancreas and (F) intestine; and *γ*‐glutamyl transferase (*γ*‐GT) in (G) hepatopancreas and (H) intestine. (I, J) One‐way ANOVA and curve estimation results. Data are presented as means ± SEM (*n* = 5). Different lowercase letters indicate significant differences among 15% fish meal treatments (*p* < 0.05); asterisks indicate significant differences compared to the 35% fish meal control (*p* < 0.05,  ^∗^ *p* < 0.01,  ^∗∗^ *p* < 0.001).

**Figure:**
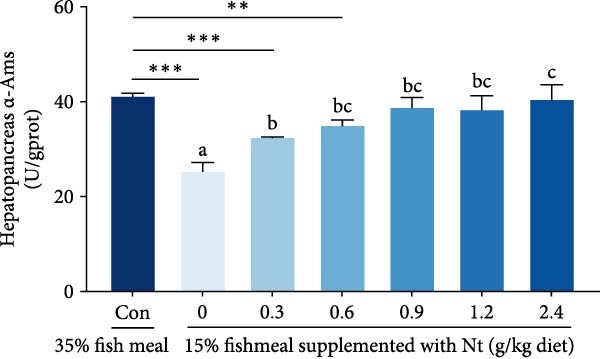
(A)

**Figure:**
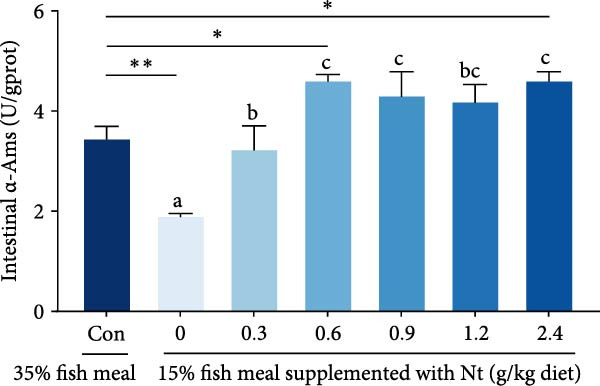
(B)

**Figure:**
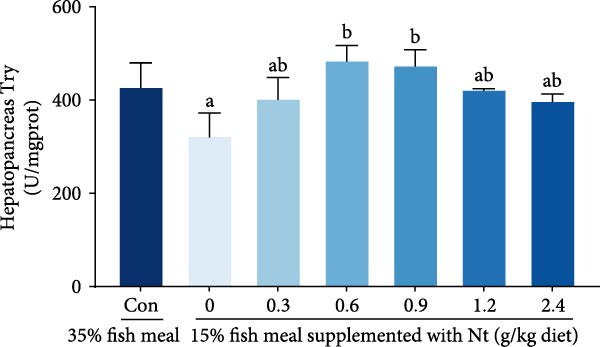
(C)

**Figure:**
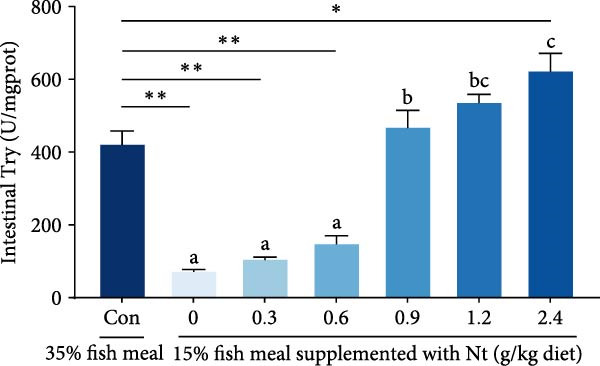
(D)

**Figure:**
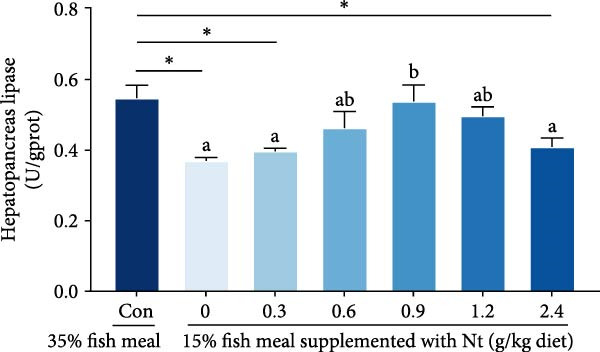
(E)

**Figure:**
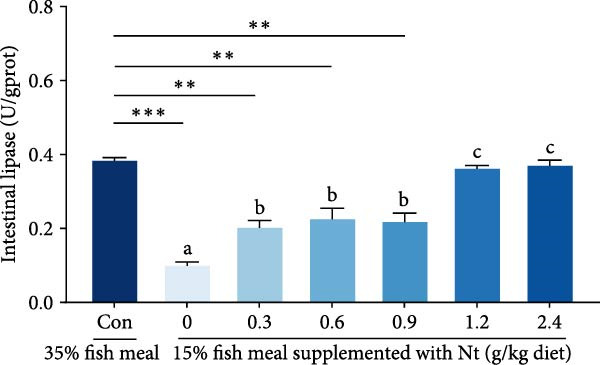
(F)

**Figure:**
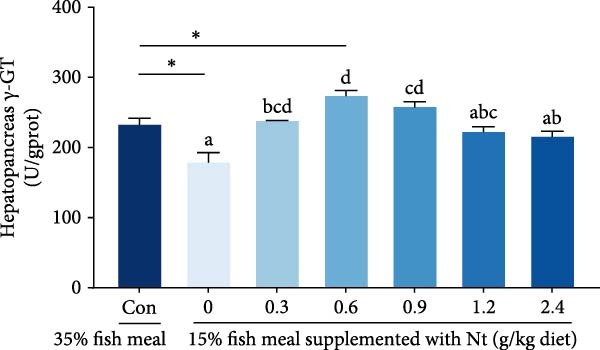
(G)

**Figure:**
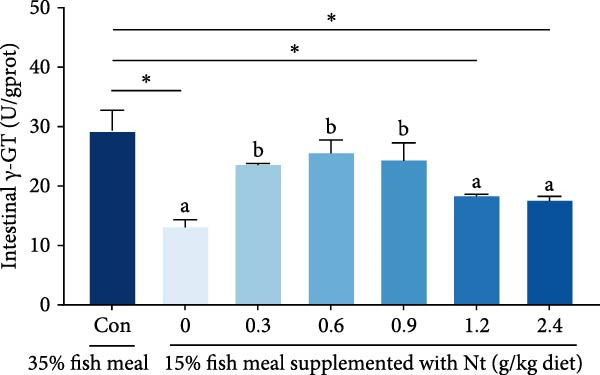
(H)

**Figure:**
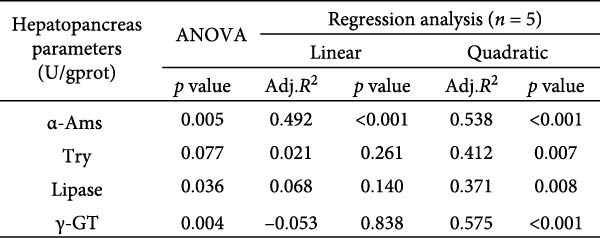
(I)

**Figure:**
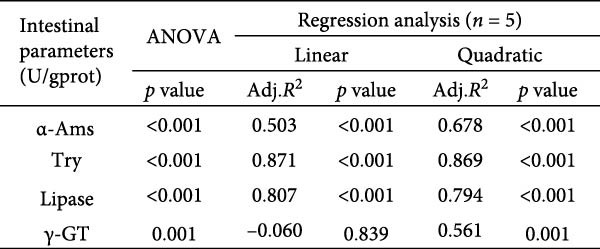
(J)

### 3.3. Growth Performance and Nutrient Utilization

Growth performance (WG, SGR, survival, and MF) was significantly impaired in the 0 g/kg nucleotide group compared to the control (*p* < 0.05), but was improved by dietary nucleotide supplementation relative to the 0 g/kg group (*p* < 0.05; Figure 3A–D). Supplementation with 0.9 g/kg nucleotide or higher restored WG, SGR, SR, and MF to levels not significantly different from the control group (*p* > 0.05). Based on WG, the optimal dietary nucleotide level was estimated as 0.85 g/kg using broken‐line regression (Figure 3E).

Figure 3Effects of dietary nucleotide supplementation on the growth performance of juvenile *Eriocheir sinensis* fed low‐fish meal diets for 56 days. (A) Weight gain (WG, %). (B) Specific growth rate (SGR, %/day). (C) Survival rate (%). (D) Molting frequency (MF, %). (E) Broken‐line regression analysis determining optimal dietary nucleotide concentration based on WG. (F) One‐way ANOVA and curve estimation results. Data are presented as means ± SEM (*n* = 5). Different lowercase letters indicate significant differences among 15% fish meal treatments (*p* < 0.05); asterisks indicate significant differences compared to the 35% fish meal control (*p* < 0.05,  ^∗^ *p* < 0.01).

**Figure:**
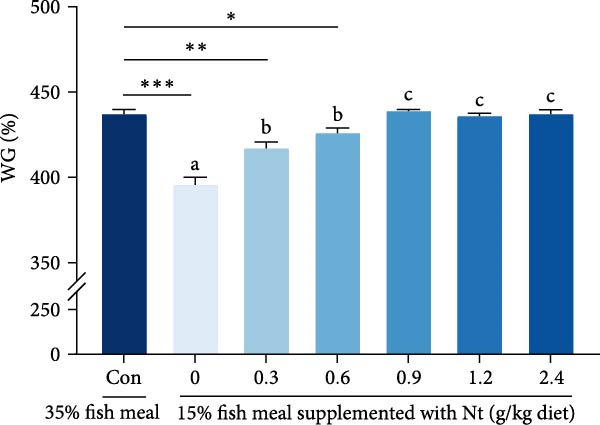
(A)

**Figure:**
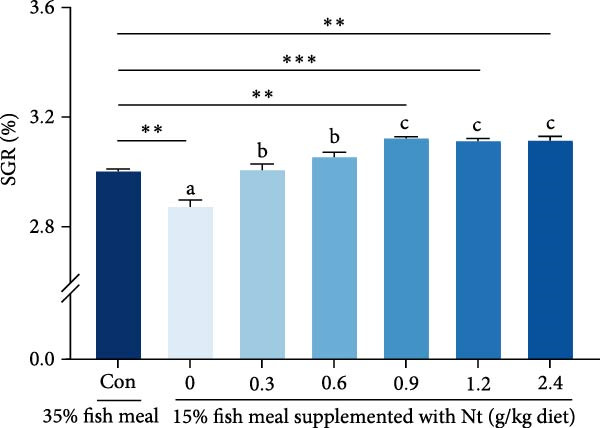
(B)

**Figure:**
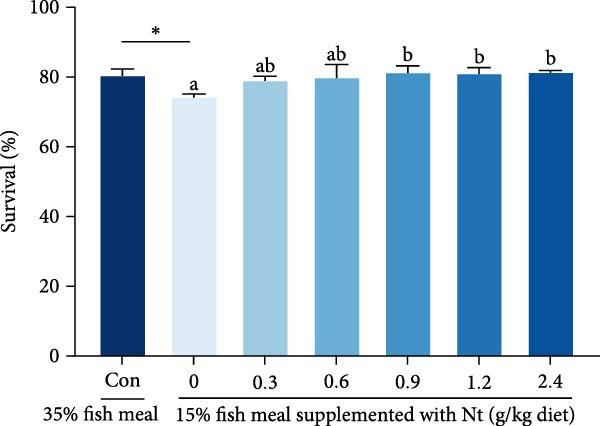
(C)

**Figure:**
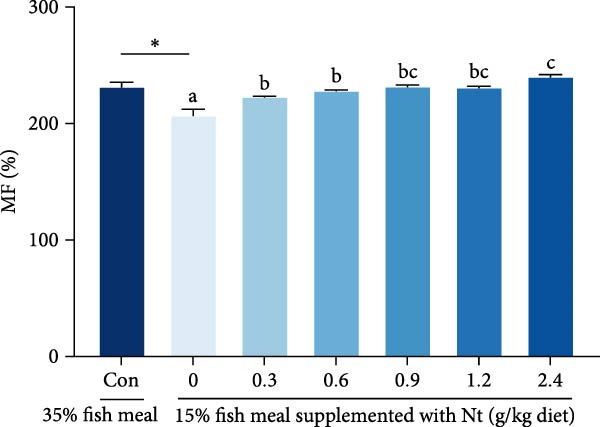
(D)

**Figure:**
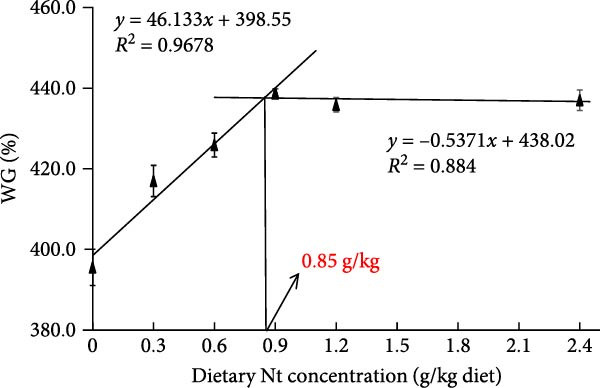
(E)

**Figure:**
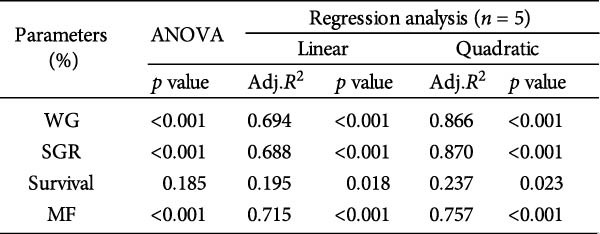
(F)

Whole‐body crude protein was lower in the 0 g/kg nucleotide group compared to the control (*p* < 0.05), but was increased by supplementation with 0.6 or 0.9 g/kg nucleotide relative to the 0 g/kg group (*p* < 0.05; Figure 4A). Feed utilization was impaired in the 0 g/kg nucleotide group compared to the control, as shown by higher FCR (*p* < 0.05) and lower PER and PR (*p* < 0.01; Figure 4B–D). Dietary nucleotide supplementation significantly improved all feed utilization parameters relative to the 0 g/kg group (lower FCR, higher PER, and PR; *p* < 0.05).

Figure 4Effects of dietary nucleotide supplementation on body composition and feed utilization in juvenile *Eriocheir sinensis* fed low‐fish meal diets. (A) Whole‐body crude protein (%). (B) Feed conversion ratio (FCR). (C) Protein efficiency ratio (PER). (D) Protein retention (PR, %). (E) One‐way ANOVA and curve estimation results. Data are presented as means ± SEM (*n* = 5). Different lowercase letters indicate significant differences among 15% fish meal treatments (*p* < 0.05); asterisks indicate significant differences compared to the 35% fish meal control (*p* < 0.05,  ^∗^ *p* < 0.01).

**Figure:**
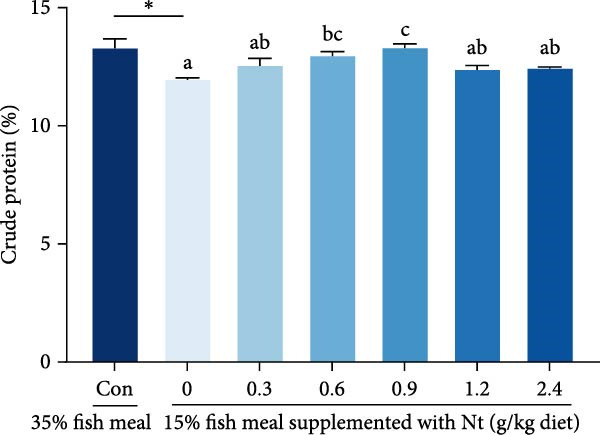
(A)

**Figure:**
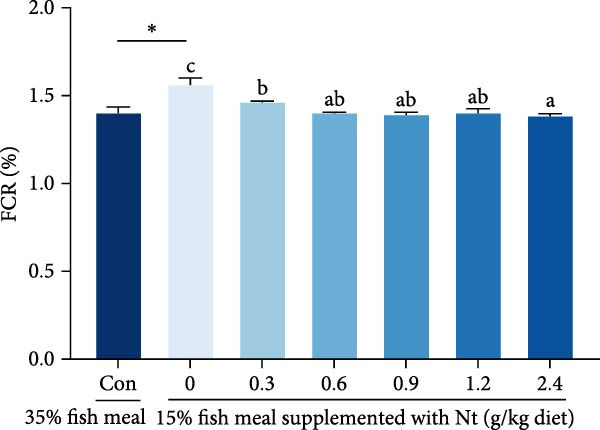
(B)

**Figure:**
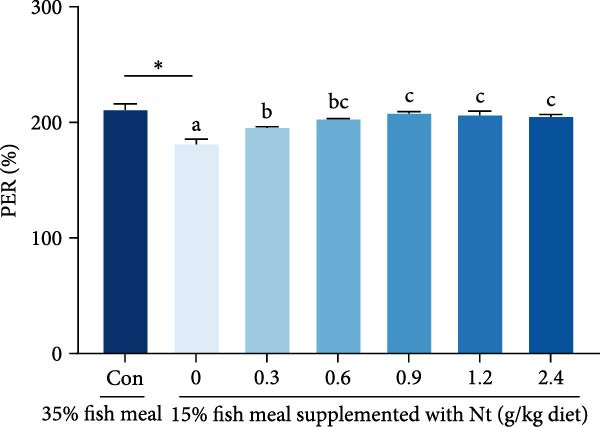
(C)

**Figure:**
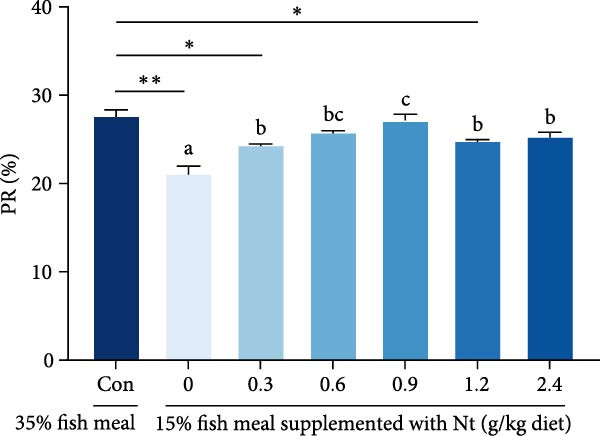
(D)

**Figure:**
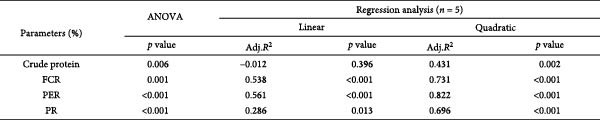
(E)

### 3.4. Intestinal Morphology and Related Gene Expression

Compared to the control group (35% fish meal, Figure 5A), the crabs fed 15% fish meal supplemented with 0 g/kg nucleotide exhibited detachment of the peritrophic membrane (Figure 5B). Supplementation with 0.9 g/kg nucleotide or higher restored the intestinal morphology to a state similar to the control group (Figure 5E–G). P38‐MAPK, EsRelish, and Bax expression levels were significantly elevated in the crabs fed 15% fish meal supplemented with 0 g/kg nucleotide compared to the control group (*p* < 0.05; Figure 5H–J). Nucleotide supplementation reduced the expression of these genes, bringing them to levels not significantly different from the control group (*p* > 0.05). Conversely, expression of the antiapoptosis gene Bcl2 tended to be increased by nucleotide supplementation, with the 0.9 g/kg dose showing significantly higher expression than the crabs fed 15% fish meal supplemented with 0 g/kg nucleotide (*p* < 0.05; Figure 5K).

Figure 5Effects of dietary nucleotide supplementation on intestinal morphology and inflammatory/apoptotic markers in juvenile *Eriocheir sinensis* fed low‐fish meal diets. (A–G) Representative H&E‐stained intestinal sections from treatment groups: (A) Control (35% fish meal); (B–G) 15% fish meal diets supplemented with 0, 0.3, 0.6, 0.9, 1.2, and 2.4 g/kg nucleotide, respectively. Arrows indicate peritrophic membrane‐epithelium relationship (a: attached; b, c: detached). Scale bars = 50 µm. (H–K) Relative intestinal mRNA expression of *p38-MAPK* (H), *EsRelish* (I), *Bax* (J), and *Bcl2* (K). (L) One‐way ANOVA and curve estimation results. Data are presented as means ± SEM (*n* = 5). Different lowercase letters indicate significant differences among 15% fish meal treatments (*p* < 0.05); asterisks indicate significant differences compared to the 35% fish meal control (*p* < 0.05,  ^∗^ *p* < 0.01).

**Figure:**
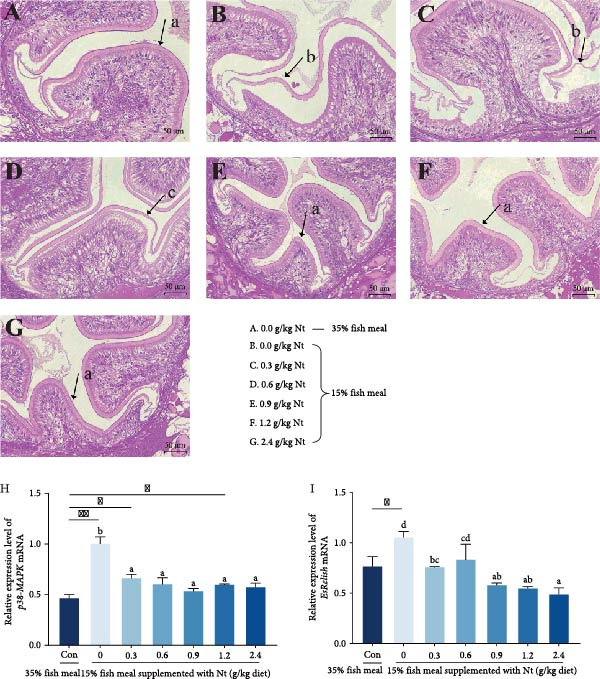

**Figure:**
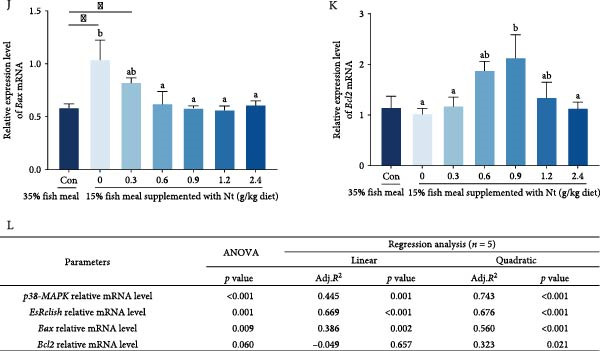

### 3.5. Hepatopancreas Transcriptome Analysis

Hepatopancreas transcriptomic profiles differed distinctly between the group fed 15% fish meal supplemented with 0 g/kg nucleotide and the group fed 15% fish meal supplemented with 0.9 g/kg nucleotide (Figure 6A). A total of 108 DEGs were identified (|log_2_FC| > 2, *p*‐adjust < 0.05; Figure 6B), 42 genes upregulated and 66 genes downregulated in the group fed 15% fish meal supplemented with 0.9 g/kg nucleotide compared to the group fed 15% fish meal supplemented with 0 g/kg nucleotide. KEGG analysis showed these DEGs were significantly enriched (*p*‐adjust < 0.05) in pathways, including glutathione metabolism, thyroid hormone synthesis, glycerophospholipid metabolism, and AMPK signaling (Figure 6D). Network analysis highlighted key DEGs (Figure 6E and Table 2). Notably, genes related to antioxidant defense (GPX2 and Gpx‐like) and phospholipid synthesis (PCYT2 and PISD) were upregulated, while HGD (tyrosine metabolism) was downregulated in the group fed 15% fish meal supplemented with 0.9 g/kg nucleotide compared to the group fed 15% fish meal supplemented with 0 g/kg nucleotide (Table 2).

Figure 6Hepatopancreas transcriptomic analysis of juvenile *Eriocheir sinensis* fed low‐fish meal diets with or without nucleotide supplementation. Comparison between 15% fish meal groups supplemented with 0 g/kg vs. 0.9 g/kg nucleotide. (A) Principal Component Analysis of transcriptome profiles. (B) Volcano plot showing differentially expressed genes (DEGs); significance thresholds: |log_2_FC| > 2, *p*‐adjust < 0.05. (C) KEGG pathway classification of DEGs by functional category. (D) KEGG pathway enrichment analysis with bubble size representing gene count and color intensity reflecting statistical significance (*p*‐adjust < 0.05). (E) Interaction network of key DEGs potentially mediating nucleotide effects. Transcriptome data obtained from *n* = 3 individuals per group.

**Figure:**
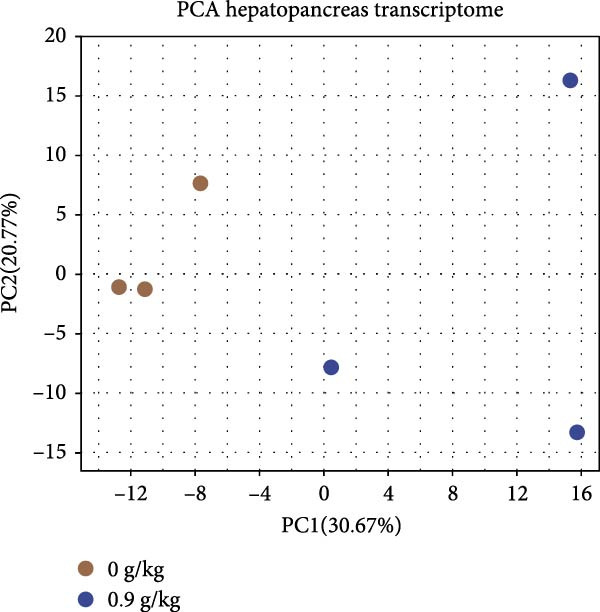
(A)

**Figure:**
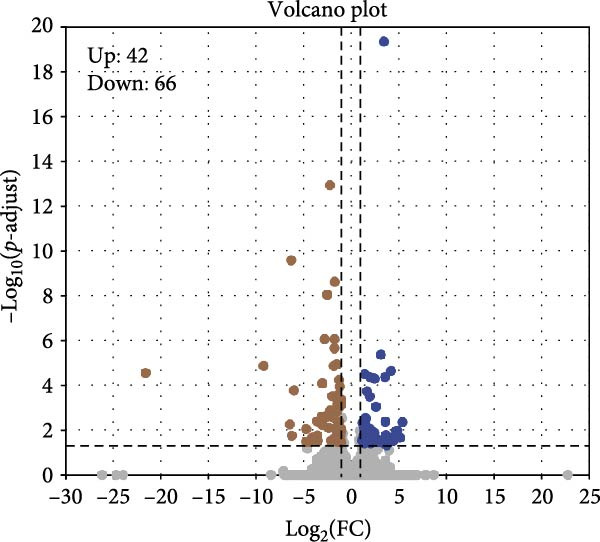
(B)

**Figure:**
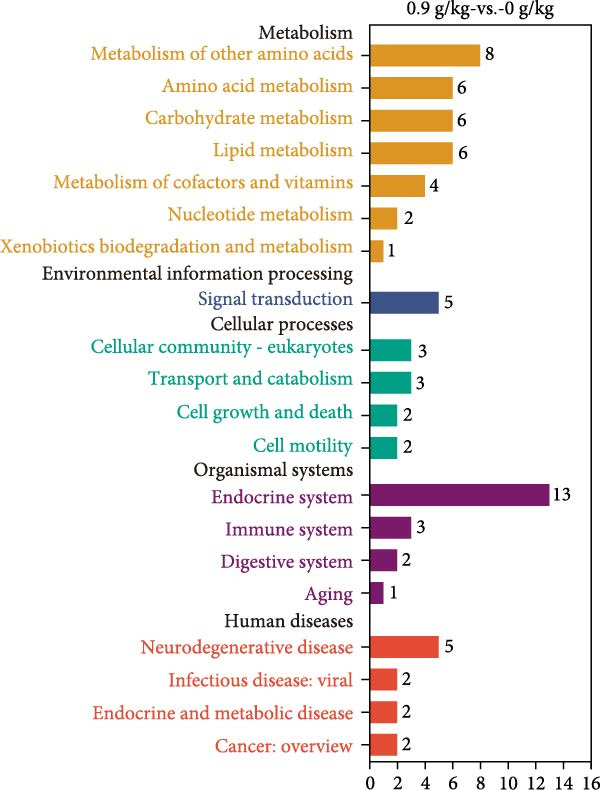
(C)

**Figure:**
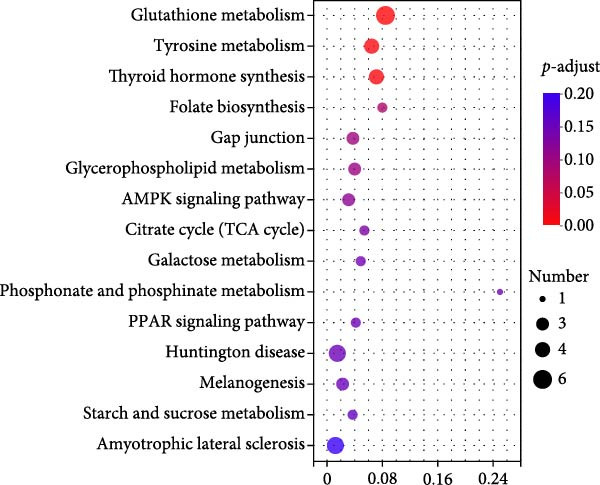
(D)

**Figure:**
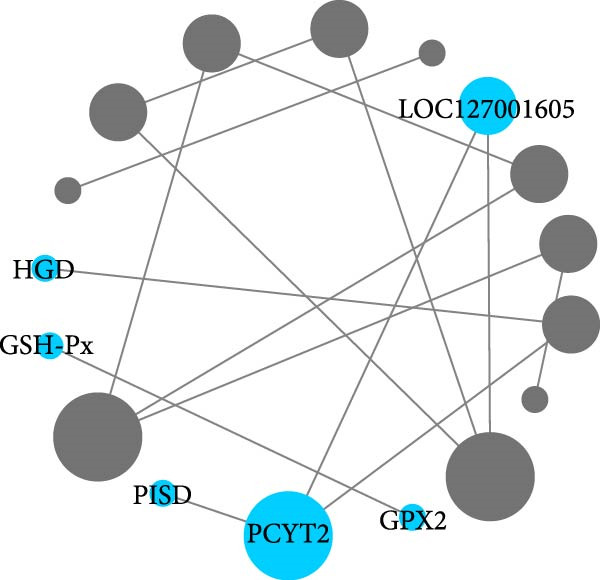
(E)

**Table 2:** Key DEGs in the hepatopancreas of juvenile *Eriocheir sinensis* comparing 15% fish meal diets supplemented with 0.9 g/kg vs. 0 g/kg nucleotide^a^.

Gene name	Gene description	Gene symbol	Log_2_ FC	Regulate
LOC127005503	Homogentisate 1,2‐dioxygenase‐like	HGD	−2.25	Down
LOC127009970	Glutathione peroxidase 2‐like, transcript variant X6	GPX2	3.11	Up
LOC126991765	Glutathione peroxidase‐like	Gpx‐like	3.53	Up
LOC127001605	Uncharacterized LOC127001605, transcript variant X1	—	1.97	Up
LOC127007745	Ethanolamine‐phosphate cytidylyltransferase‐like, transcript variant X1	PCYT2	1.54	Up
LOC127005825	Phosphatidylserine decarboxylase proenzyme, mitochondrial‐like, transcript variant X1	PISD	2.02	Up

^a^Genes potentially mediating nucleotide effects identified via network analysis (Figure 6E). Log_2_ FC represents the log_2_ fold change (0.9 g/kg nucleotide group relative to 0 g/kg nucleotide group). *p*‐adjust is the adjusted *p*‐value for differential expression.

### 3.6. Intestinal Microbiota Diversity, Composition, and Interaction Networks

Alpha diversity indices (Shannon, ACE, and Chao) were significantly higher in the 0.9 g/kg nucleotide group compared to the 0 g/kg nucleotide group (*p* < 0.01), with the Control group showing intermediate values (Figure 7A,C,D). PCoA revealed distinct clustering and significant structural differences among the three groups (*p* < 0.001; Figure 7E). Specificity‐occupancy analysis identified substantially more key bacterial species in the 0.9 g/kg nucleotide group (*n* = 94) compared to both control (*n* = 13) and 0 g/kg nucleotide (*n* = 18) groups (Supporting Information 1: Figure S2A). Among these key species, Proteobacteria, Actinobacteriota, and Verrucomicrobiota were predominant phyla in the 0.9 g/kg nucleotide group (Supporting Information 1: Figure S2B). Characteristic genera included *Ilumatobacter* and *Leucobacter* for the control group, *Dechloromonas* for the 0 g/kg nucleotide group, and multiple genera such as *Tabrizicola*, *Nocardia*, and *Gemmata* for the 0.9 g/kg nucleotide group (Table 3). Microbial co‐occurrence network structures also differed among treatments (Figure 7G), with the 0 g/kg nucleotide group displaying the lowest proportion of positive correlations. The 0.9 g/kg nucleotide group showed an intermediate proportion of positive correlations and the highest average clustering coefficient among all groups (Figure 7H).

Figure 7Effects of dietary nucleotide supplementation on intestinal microbiota of juvenile *Eriocheir sinensis*. Comparisons among Control (35% fish meal), low‐fish meal (15% fish meal +0 g/kg nucleotide), and nucleotide‐supplemented low‐fish meal (15% fish meal +0.9 g/kg nucleotide) groups. (A–D) Alpha diversity indices: Shannon (A), Simpson (B), ACE (C), and Chao (D). (E) Principal coordinate analysis (PCoA) showing microbial community dissimilarities with significance values from permutational multivariate analysis. (F) Specificity‐occupancy plot identifying key bacterial genera (bubble size indicates relative abundance, colors represent phyla). (G) Microbial co‐occurrence networks showing positive (red) and negative (green) correlations between taxa. (H) Network topological properties. Data derived from *n* = 5 individuals per group. Asterisks indicate significant differences between groups (*p* < 0.05,  ^∗^ *p* < 0.01,  ^∗∗^ *p* < 0.001).

**Figure:**
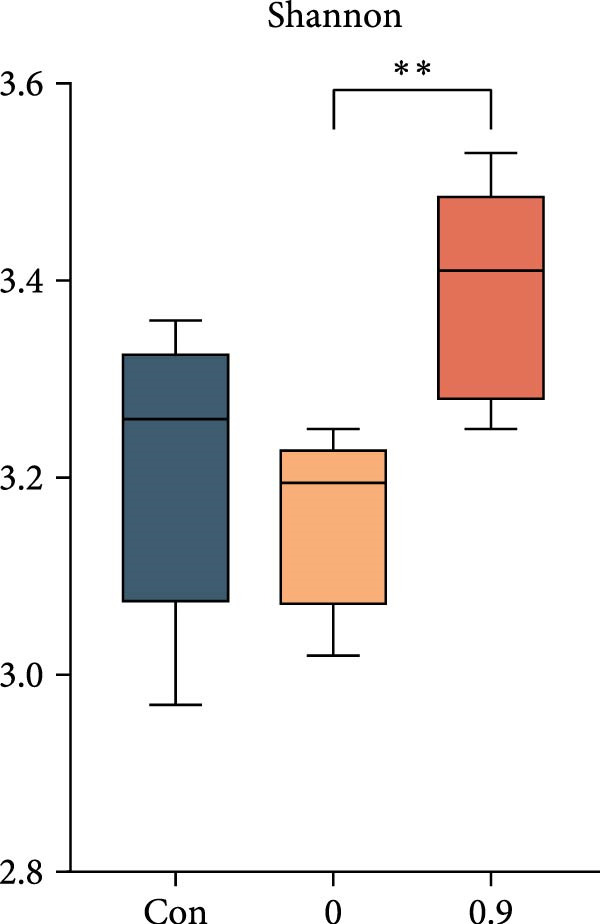
(A)

**Figure:**
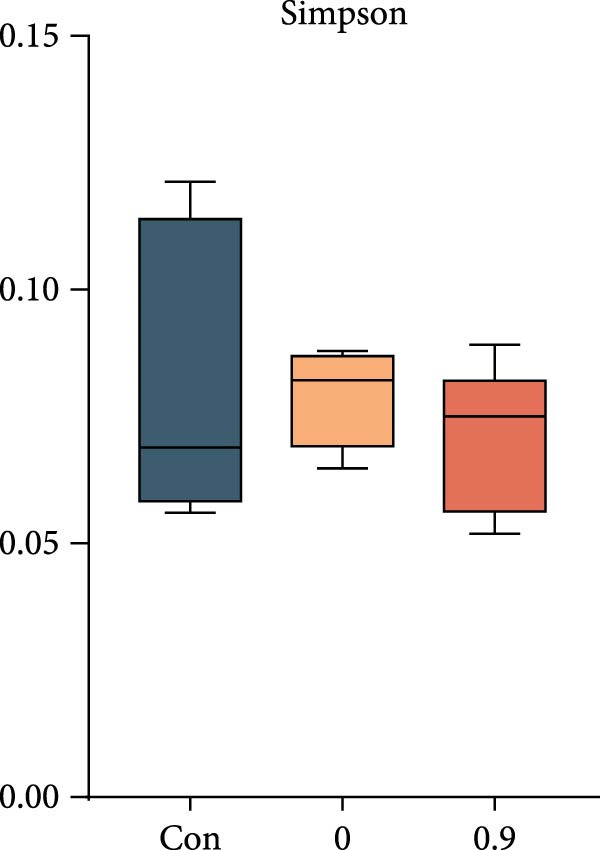
(B)

**Figure:**
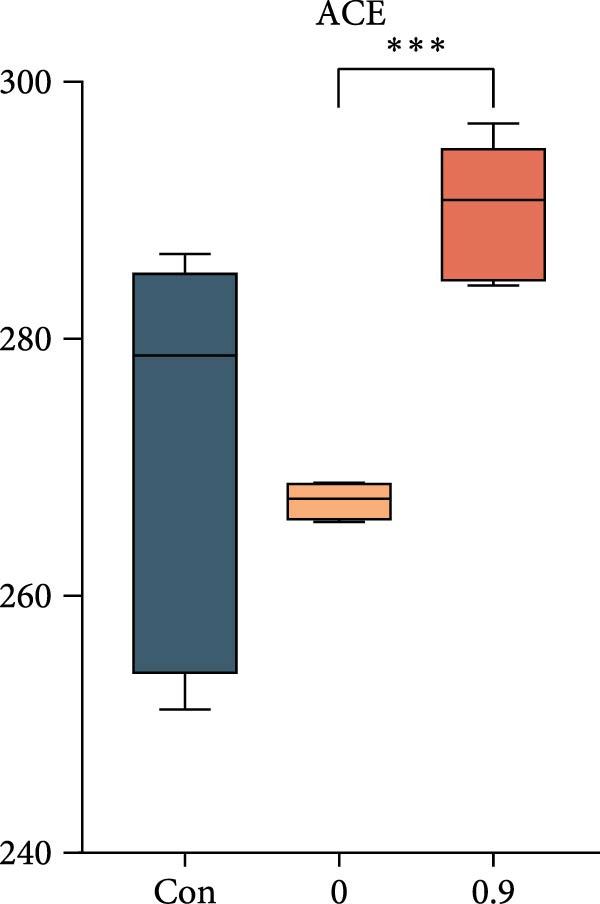
(C)

**Figure:**
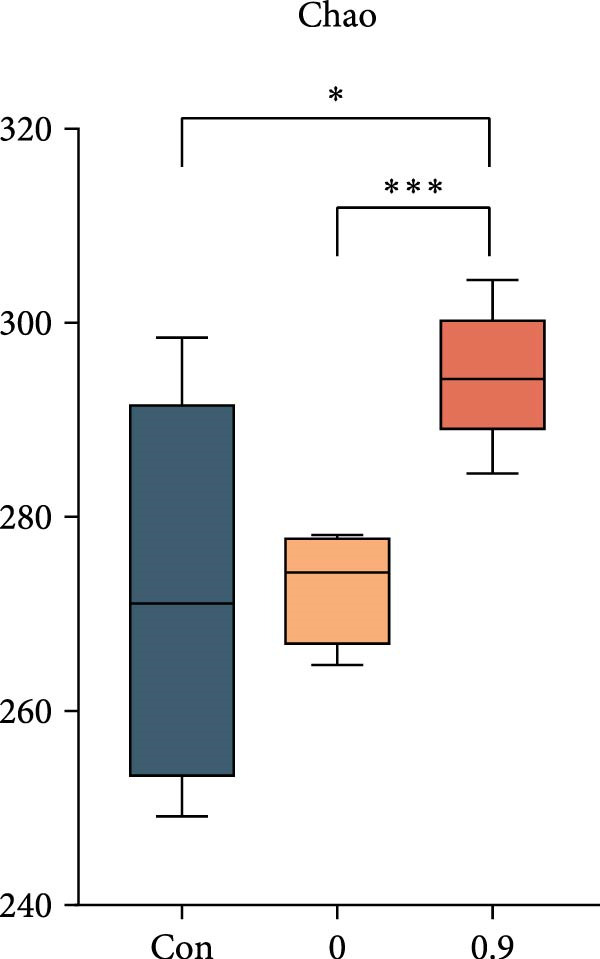
(D)

**Figure:**
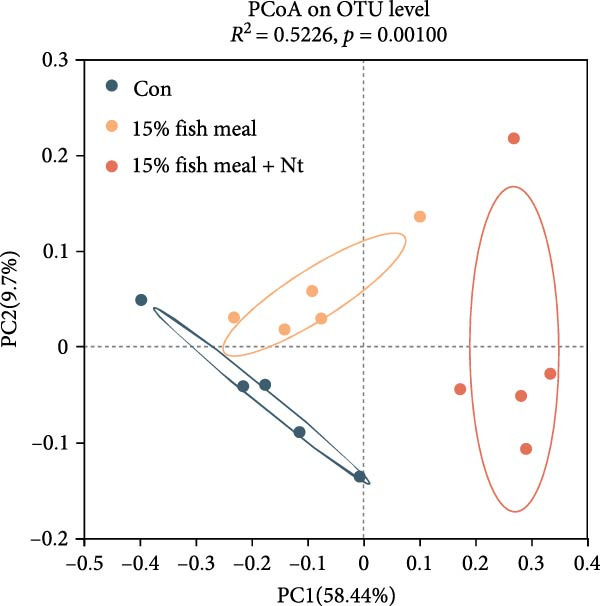
(E)

**Figure:**
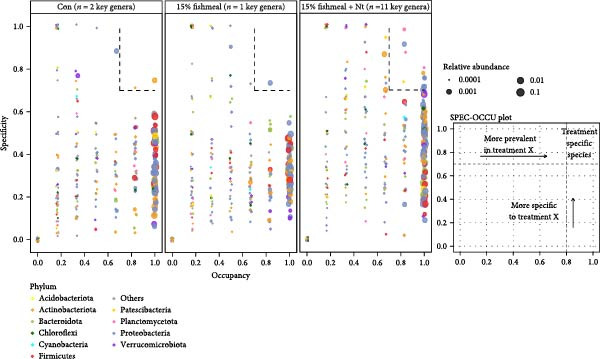
(F)

**Figure:**
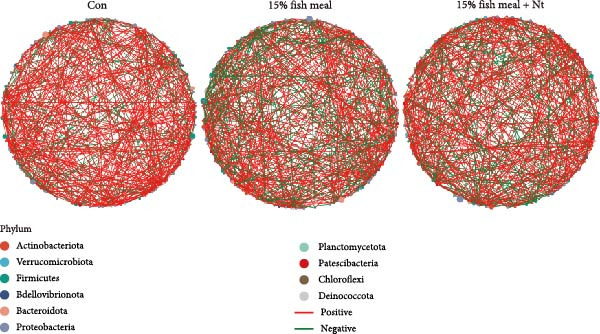
(G)

**Figure:**
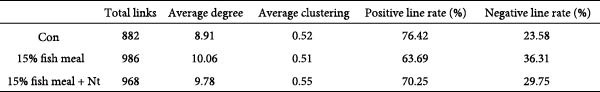
(H)

**Table 3:** Specificity and occupancy scores of key bacterial genera characterizing the gut microbiota of juvenile *Eriocheir sinensis* across different dietary treatments^a^.

Group	Genus	Phylum	Specificity	Occupancy
Con	*Ilumatobacter*	Actinobacteriota	0.76	1
Con	*Leucobacter*	Actinobacteriota	0.71	0.83
0‰ Nt	*Dechloromonas*	Proteobacteria	0.73	0.83
0.9‰ Nt	*Tabrizicola*	Proteobacteria	0.71	1
0.9‰ Nt	*Candidatus_Berkiella*	Proteobacteria	0.71	1
0.9‰ Nt	*Unclassified_Rickettsiales*	Proteobacteria	0.92	0.83
0.9‰ Nt	*Unclassified_Sphingomonadaceae*	Proteobacteria	0.76	1
0.9‰ Nt	*Rubellimicrobium*	Proteobacteria	0.72	1
0.9‰ Nt	*Nocardia*	Actinobacteriota	0.77	1
0.9‰ Nt	*Candidatus_Ovatusbacter*	Proteobacteria	0.76	1
0.9‰ Nt	*Gemmata*	Planctomycetota	0.98	0.83
0.9‰ Nt	*Unclassified_norank_c__OM190*	Planctomycetota	0.81	1
0.9‰ Nt	*Unclassified_Isosphaeraceae*	Planctomycetota	0.88	0.83
0.9‰ Nt	*Unclassified_Candidatus_Kerfeldbacteria*	Patescibacteria	0.74	0.83

^a^Dietary treatments: Con = control diet containing 35% fish meal; 0‰ Nt = 15% fish meal diet supplemented with 0 g/kg nucleotide; 0.9‰ Nt = 15% fish meal diet supplemented with 0.9 g/kg nucleotide.

### 3.7. RDA of Gut Microbiota and Host Physiological Parameters

To explore potential relationships between microbiota composition and host physiological parameters, RDA was conducted following principal component analysis of host indicators. The first principal component (PC1) accounted for 23.1% of host physiological variance and showed statistical correlation with growth performance metrics, including weight gain, specific growth rate, PR, and survival (*r* > 0.71 for all; Figure 8B, Supporting Information 1: Table S3). When using host physiological PCs as constraining variables for microbial community analysis, only PC1 demonstrated a significant statistical association with gut microbiota structure (*p* = 0.007, Supporting Information 1: Table S4). The RDA ordination plot illustrates this linkage, displaying distinct clustering of the treatment groups (Figure 8A). The group fed 15% fish meal without nucleotides appeared separated from the control group (Con, 35% fish meal). In comparison, the nucleotide‐supplemented group (15% fish meal) clustered more proximally to the control group, particularly along the axis corresponding to PC1.

Figure 8Relationship between host physiological status and gut microbiota structure in juvenile *Eriocheir sinensis*. Comparison among control (35% fish meal), low‐fish meal (15% fish meal, 0 g/kg nucleotide), and nucleotide‐supplemented low‐fish meal (15% fish meal +0.9 g/kg nucleotide) groups. (A) Redundancy analysis (RDA) ordination plot showing gut microbial communities constrained by host physiological principal components (PCs). Individual samples are colored by treatment group within 95% confidence ellipses; red arrows show constraining vectors PC1–PC5. Axis labels indicate the percentage of constrained variance explained. PC1 is significantly associated with microbial community structure (*p* = 0.007). (B) Pearson correlation coefficients between PC1 scores and selected host physiological indicators with strong correlations (|*r*| > 0.5), colored by functional category.

**Figure:**
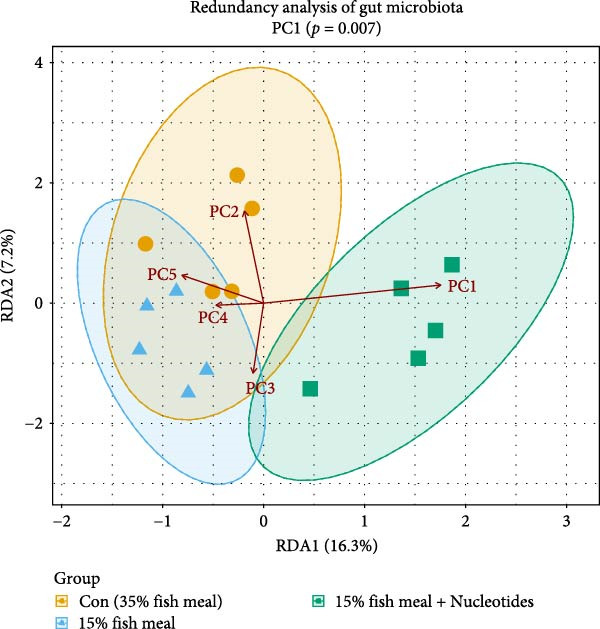
(A)

**Figure:**
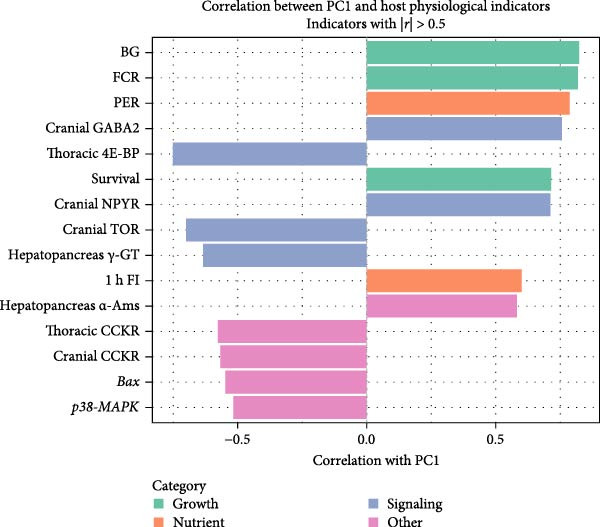
(B)

## 4. Discussion

Dietary nucleotides improved growth and physiological health in juvenile *E. sinensis* fed low‐fish meal diets. At appropriate inclusion (e.g., 0.9 g/kg), multiple endpoints—including intake and growth—were restored to levels not significantly different from the high‐fish meal control, while broken‐line regression indicated optima of approximately 0.76 g/kg for intake and approximately 0.85 g/kg for growth. These effects likely involve enhanced feed intake, improved digestive capacity, preserved intestinal integrity, strengthened hepatopancreatic antioxidant capacity, and microbiota restructuring aligned with host performance.

### 4.1. Enhanced Feed Intake as the Primary Driver of Improved Performance

A key nucleotide effect was significantly improved feed intake, likely contributing to enhanced growth. Supplementation dose‐dependently increased feed consumption, with 0.9 g/kg restoring intake to control levels. Broken‐line regression estimated the optimum for intake at 0.76 g/kg. This value, slightly below 0.9 g/kg, achieving statistical parity with controls, distinguishes mathematical optimization from biological equivalence and suggests a response plateau at higher levels. Enhanced intake directly addresses the poor palatability of plant‐rich diets for carnivorous crustaceans [32, 33]. Furthermore, modulated expression of appetite‐related genes (favoring orexigenic signals) suggests central regulation complements peripheral chemosensation. The taste enhancers like IMP and GMP, abundant in fish meal but low in plant sources, likely mediate these chemosensory effects [34, 35]. However, responses are species‐specific as nucleotides can increase intake and growth in red sea bream [17], but improve growth without affecting intake in hybrid striped bass [22]. This highlights that different primary mechanisms (e.g., intake stimulation vs. metabolic efficiency) may operate across species, influencing physiological outcomes. Critically, this enhanced consumption directly impacts nutrient availability for downstream digestive processes.

### 4.2. Improved Digestive Physiology and Nutrient Utilization

Beyond increasing intake, dietary nucleotides appear to enhance digestive efficiency. Elevated activities of *α*‐Ams, Try, and lipase in the hepatopancreas and intestine are consistent with better feed utilization, but we view these changes as partly substrate‐driven and, therefore, complementary to rather than exclusively causal for performance gains. A direct nucleotide‐mediated activation of digestive pathways remains plausible [36]. By improving enzymatic capacity, crabs may better cope with the poor digestibility of plant ingredients [37]. Increased hepatopancreatic *γ*‐GT further aligns with enhanced amino‐acid transport [38, 39], supporting tissue synthesis and growth. While nutrient‐sensing feedback could reinforce appetite and digestion, efficient utilization ultimately depends on the structural integrity of the intestine.

### 4.3. Preservation of Intestinal Integrity and Function

Under low‐fish meal stress, nucleotide supplementation preserved intestinal architecture—notably peritrophic membrane attachment—and attenuated inflammatory/apoptotic signaling, as indicated by our histology and gene‐expression readouts and in line with reports that nucleotides support epithelial integrity under nutritional challenge [40, 41]. The pro‐inflammatory and pro‐apoptotic signaling milieu appeared attenuated, as indicated by reduced expression of p38‐MAPK, Relish, and Bax together with increased Bcl2, a pattern consistent with observations in other species under dietary challenge [42–44]. Such barrier preservation plausibly facilitates nutrient absorption and supports a milieu permissive to beneficial microbial communities, thereby contributing to improved feed utilization and growth; however, these associations remain correlative and do not by themselves establish direct causality [45, 46].

### 4.4. Hepatopancreatic Metabolic Adaptation and Antioxidant Defense

Once nutrients are absorbed through an intact intestine, effective assimilation requires efficient subsequent metabolic processing and stress management, primarily processed by the hepatopancreas. Enhanced antioxidant defense capacity emerged as a prominent hepatopancreatic effect in nucleotide‐supplemented crabs. The activation of the glutathione metabolism pathway and upregulation of glutathione peroxidase genes (GPX2 and Gpx‐like) suggest the enhancement of resilience against oxidative stress induced by antinutritional factors present in the plant‐based low‐fish meal diets [47, 48]. This antioxidant enhancement may occur if nucleotides serve as precursors for NADPH production via the pentose phosphate pathway (critical for glutathione regeneration) or potentially through nucleotide‐mediated activation of redox‐sensitive transcription factors regulating antioxidant response elements [49, 50]. Additional transcriptomic changes, such as upregulated genes involved in membrane biosynthesis (*PCYT2* and *PISD*) and the downregulated *HGD* gene (involved in tyrosine catabolism), indicate that metabolic adaptations favor anabolic processes [51, 52]. Specifically, the downregulation of *HGD* suggests a metabolic shift toward amino acid conservation, allowing preferential utilization of amino acids for protein synthesis rather than catabolism [53]. These hepatopancreatic modifications represent metabolic adaptations to improve nutrient processing and utilization. The improved metabolism enhances nutrient acquisition, which may contribute to the observed growth performance.

### 4.5. Beneficial Modulation of Gut Microbiota and Host–Microbe Interactions

In addition to direct effects on host tissues like the intestine and hepatopancreas, dietary components profoundly influence the gut microbiota, which in turn impacts host physiology. Nucleotide supplementation significantly restructured the gut microbiota, correlating with improved host performance. Enhanced alpha diversity and modified network properties can improve ecosystem stability to benefit hosts [54]. Taxonomically, the low‐fish meal group without nucleotide supplementation was characterized by the genus *Dechloromonas*, reflecting microbial adaptation to the challenging dietary conditions [55, 56]. In contrast, the nucleotide‐supplemented group significantly increased the number of beneficial bacterial species. These taxa, such as *Tabrizicola*, *Nocardia*, and *Gemmata*, have been associated in previous studies with gut homeostasis or the degradation of complex organic compounds [57, 58], suggesting a potential functional shift that could support the host’s ability to process the plant‐based diet. This aligns with the observed enrichment of Verrucomicrobiota, which are also known for degrading complex polysaccharides [59]. Beyond compositional shifts, the microbial co‐occurrence network in nucleotide‐fed crabs displayed a higher clustering coefficient. In an ecological context, this network feature has been associated with increased community stability and resistance to perturbation [60]. The more clustered network structure suggests that nucleotides may foster a functionally integrated microbiota.

Experimental evidence confirms a crucial role for gut microbes in mediating nucleotide benefits. For instance, zebrafish receiving microbiota transplants from nucleotide‐fed donors can reduce energy expenditure and enhance growth [61]. While specific microbial mechanisms differ between fish and crustaceans, we observed strong evidence that nucleotide supplementation can mediate microbiota benefits in the crab intestine. The precise functional contributions of specific taxa require further investigation in this crab model, but the microbial shift strongly suggests a positive physiological response to nucleotide supplementation.

The pronounced nucleotide efficacy in the low‐fish meal diet supports their “conditional essentiality” [62], where endogenous synthesis becomes insufficient under stress or dietary challenge. Low‐fish meal diets increase metabolic demand while reducing exogenous nucleotide supply. Dietary nucleotides become conditionally essential, and supplementation must be supplied to fill this gap. This context‐dependency explains why nucleotides boost growth in low‐fish meal diets but offer less benefit when fish meal content is high [15]. A limitation of this study, however, is the use of a nucleotide mixture. This approach, while effective, prevents us from identifying the specific roles of individual nucleotides. Future research should elucidate causal microbiota–host links and evaluate individual nucleotide roles to refine the supplementation protocol.

## 5. Conclusions

**Figure 9:**
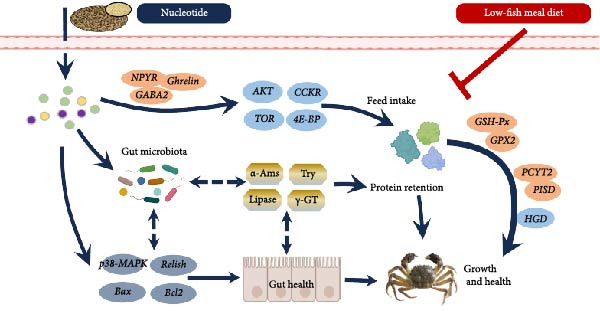
Possible mechanisms of dietary nucleotides in improving the growth and health of Chinese mitten crabs (*Eriocheir sinensis*) fed low‐fish meal diets.

Replacing fish meal with plant‐based protein impaired *E. sinensis* performance, but dietary nucleotide supplementation effectively restored growth and physiological health. Based on broken‐line regression of weight gain, the optimal dietary nucleotide level for juvenile *E. sinensis* fed this low‐fish meal diet was estimated at approximately 0.85 g/kg. This supplementation stimulated feed intake, enhanced digestion, preserved gut integrity, bolstered antioxidant defenses, and favorably modulated gut microbiota (Figure 9). These findings validate dietary nucleotides as a valuable tool to develop sustainable, low‐fish meal feeds for Chinese mitten crab aquaculture.

## Conflicts of Interest

The authors declare that Erchao Li and Liqiao Chen are members of the Editorial Board of Aquaculture Nutrition. However, Erchao Li and Liqiao Chen had no involvement in the peer review or editorial decision‐making process related to this manuscript. The other authors declare no conflicts of interest.

## Funding

This work was supported by grants from the National Key Research and Development Program of China (Grant 2023YFD2402000), the Agriculture Research System of Shanghai, China (Grant 202404), and the Agriculture Research System of China of MOF and MARA (Grant CARS‐48).

## Supporting Information

Additional supporting information can be found online in the Supporting Information section.

## Supporting information


**Supporting Information 1** Table S1. Nucleotide composition of experimental diets (mg/100g dry matter). Table S2. Primer pair sequences of the genes used for real‐time PCR (qPCR). Table S3. Summary of principal component analysis of host physiological indicators. Table S4. Significance of marginal effects of host physiological principal components on gut microbiota structure in redundancy analysis. Figure S1. Effects of dietary nucleotide supplementation on whole‐body crude lipid and crude ash of juvenile *Eriocheir sinensis* fed low‐fish meal diets. Figure S2 Identification and phylum distribution of key bacterial species characterizing the gut microbiota of juvenile *E. sinensis* across different dietary treatments.

**Supporting Information 2** File S2. ARRIVE 2.0 Author Checklist completed for this study.

## Data Availability

The data that support the findings of this study are available from the corresponding author upon reasonable request.

## References

[1] Tacon A. G. J. and Metian M. , Feed Matters: Satisfying the Feed Demand of Aquaculture, Reviews in Fisheries Science & Aquaculture. (2015) 23, no. 1, 1–10, 10.1080/23308249.2014.987209, 2-s2.0-84929251881.

[2] FAO , The State of World Fisheries and Aquaculture 2024, 2024, FAO.

[3] Naylor R. L. , Hardy R. W. , and Bureau D. P. , et al.Feeding Aquaculture in an Era of Finite Resources, Proceedings of the National Academy of Sciences. (2009) 106, no. 36, 15103–15110, 10.1073/pnas.0905235106, 2-s2.0-70349338795. PMC2741212 19805247

[4] Shepherd C. J. and Jackson A. J. , Global Fishmeal and Fish-Oil Supply: Inputs, Outputs and Marketsa, Journal of Fish Biology. (2013) 83, no. 4, 1046–1066, 10.1111/jfb.12224, 2-s2.0-84884966731. 24090562

[5] Stickney R. R. and McVey J. P. , Responsible Marine Aquaculture, 2002, 1st edition, CABI Publishing, UK, 10.1079/9780851996042.0000.

[6] Hussain S. M. , Bano A. A. , and Ali S. , et al.Substitution of Fishmeal: Highlights of Potential Plant Protein Sources for Aquaculture Sustainability, Heliyon. (2024) 10, no. 4, 10.1016/j.heliyon.2024.e26573, e26573. 38434023 PMC10906437

[7] Tantikitti C. , Feed Palatability and the Alternative Protein Sources in Shrimp Feed, 2014.

[8] Daniel N. , A Review on Replacing Fish Meal in Aqua Feeds Using Plant Protein Sources, International Journal of Fisheries and Aquatic Studies. (2018) 6, 164–179.

[9] Qu K. , He G. , and Shi M. , et al.Effects of Compound Feed Attractants on the Growth Rate, Feed Consumption, Intestinal Histology, Protein Synthesis, and Immune Response of Black Tiger Shrimp (*Penaeus monodon*), Animal Feed Science and Technology. (2024) 311, 10.1016/j.anifeedsci.2024.115952, 115952.

[10] De La Higuera M. , Houlihan D. , Boujard T. , and Jobling M. , Effects of Nutritional Factors and Feed Characteristics on Feed Intake, Food Intake in Fish, 2001, Wiley, 250–268, 10.1002/9780470999516.ch11.

[11] Glencross B. D. , Baily J. , Berntssen M. H. G. , Hardy R. , MacKenzie S. , and Tocher D. R. , Risk Assessment of the use of Alternative Animal and Plant Raw Material Resources in Aquaculture Feeds, Reviews in Aquaculture. (2020) 12, no. 2, 703–758, 10.1111/raq.12347, 2-s2.0-85065186006.

[12] Gemede H. F. and Ratta N. , Anti Dietary Factors in Plant Foods: Potential Health Benefits and Adverse Effects, 2018.

[13] Dhar V. , Singh S. K. , Narsale S. A. , Debbarma S. , Saikia P. , and Yirang Y. , Fishmeal Substitutions and Their Implications for Aquatic Animal Immune and Gut Function: A Review, Comparative Immunology Reports. (2024) 7, 10.1016/j.cirep.2024.200171, 200171.

[14] Hossain M. S. , Small B. C. , Kumar V. , and Hardy R. , Utilization of Functional Feed Additives to Produce Cost-Effective, Ecofriendly Aquafeeds High in Plant-Based Ingredients, Reviews in Aquaculture. (2024) 16, no. 1, 121–153, 10.1111/raq.12824.

[15] de Cruz C. R. , Yamamoto F. Y. , Ju M. , Chen K. , Velasquez A. , and Gatlin D. M.III, Efficacy of Purified Nucleotide Supplements on the Growth Performance and Immunity of Hybrid Striped Bass Morone Chrysops x *morone saxatilis* , Fish & Shellfish Immunology. (2020) 98, 868–874, 10.1016/j.fsi.2019.11.046. 31751660

[16] Peng M. , Xu W. , Ai Q. , Mai K. , Liufu Z. , and Zhang K. , Effects of Nucleotide Supplementation on Growth, Immune Responses and Intestinal Morphology in Juvenile Turbot Fed Diets with Graded Levels of Soybean Meal (*Scophthalmus maximus* L.), Aquaculture. (2013) 392–395, 51–58, 10.1016/j.aquaculture.2013.02.002, 2-s2.0-84874398940.

[17] Hossain M. S. , Koshio S. , Ishikawa M. , Yokoyama S. , and Sony N. M. , Dietary Effects of Adenosine Monophosphate to Enhance Growth, Digestibility, Innate Immune Responses and Stress Resistance of Juvenile Red Sea Bream, Pagrus Major, Fish & Shellfish Immunology. (2016) 56, 523–533, 10.1016/j.fsi.2016.08.009, 2-s2.0-84982144236. 27514786

[18] Song J.-W. , Lim S.-J. , and Lee K.-J. , Effects of Dietary Supplementation of Inosine Monophosphate on Growth Performance, Innate Immunity and Disease Resistance of Olive Flounder (*Paralichthys olivaceus*), Fish & Shellfish Immunology. (2012) 33, no. 4, 1050–1054, 10.1016/j.fsi.2012.07.011, 2-s2.0-84866759346. 22986588

[19] Amaliah N. , Mahendradatta M. , Zainal Z. , and Salengke S. , Trends in Natural Flavor Enhancer: A Review on Umami Compounds, BIO Web of Conferences. (2024) 96, 10.1051/bioconf/20249601013, 01013.

[20] Hossain M. S. , Koshio S. , Ishikawa M. , Yokoyama S. , Sony N. M. , and Islam M. J. , Fishmeal Replacement by Soya Protein Concentrate With Inosine Monophosphate Supplementation Influences Growth, Digestibility, Immunity, Blood Health, and Stress Resistance of Red Sea Bream, Pagrus Major, Fish Physiology and Biochemistry. (2019) 45, no. 2, 613–629, 10.1007/s10695-018-0581-2, 2-s2.0-85055681206. 30367428

[21] Hossain M. S. , Koshio S. , and Kestemont P. , Recent Advances of Nucleotide Nutrition Research in Aquaculture: A Review, Reviews in Aquaculture. (2020) 12, no. 2, 1028–1053, 10.1111/raq.12370.

[22] Li P. , Lewis D. H. , and Gatlin D. M.III, Dietary Oligonucleotides From Yeast RNA Influence Immune Responses and Resistance of Hybrid Striped Bass (Morone Chrysops × *Morone saxatilis*) to Streptococcus Iniae Infection, Fish & Shellfish Immunology. (2004) 16, no. 5, 561–569, 10.1016/j.fsi.2003.09.005, 2-s2.0-2442559189. 15110330

[23] Welker T. L. , Lim C. , Yildirim-Aksoy M. , and Klesius P. H. , Effects of Dietary Supplementation of a Purified Nucleotide Mixture on Immune Function and Disease and Stress Resistance in Channel Catfish, Ictalurus Punctatus: Effects of Purified Nucleotide Mixture on Immunity, Aquaculture Research. (2011) 42, no. 12, 1878–1889, 10.1111/j.1365-2109.2010.02794.x, 2-s2.0-81255149449.

[24] Li P. , Gatlin D. M.III, and Neill W. H. , Dietary Supplementation of a Purified Nucleotide Mixture Transiently Enhanced Growth and Feed Utilization of Juvenile Red Drum, *sciaenops ocellatus* , Journal of the World Aquaculture Society. (2007) 38, no. 2, 281–286, 10.1111/j.1749-7345.2007.00096.x, 2-s2.0-34249782878.

[25] Liu X. , Zou D. , and Wang Y. , et al.Replacement of Fish Meal with Cottonseed Protein Concentrate in Chinese Mitten Crab (*Eriocheir sinensis*): Nutrient Digestibility, Growth Performance, Free Amino Acid Profile, and Expression of Genes Related to Nutrient Metabolism, Animal Nutrition. (2024) 17, 447–462, 10.1016/j.aninu.2024.02.001. 38846720 PMC11153942

[26] Yao W. , Zhang C. , and Mao H. , et al.Effects of Dietary Defatted Black Soldier Fly (*Hermetia illucens*) Larvae Meal Substituting Fish Meal on Growth, Antioxidative Capacity, Immunity, Intestinal Histology and Microbiota of Juvenile Chinese Mitten Crab (*Eriocheir sinensis*), Aquaculture Reports. (2024) 38, 10.1016/j.aqrep.2024.102302, 102302.

[27] Vogt G. , Functional Cytology of the Hepatopancreas of Decapod Crustaceans, Journal of Morphology. (2019) 280, no. 9, 1405–1444, 10.1002/jmor.21040, 2-s2.0-85068893844. 31298794

[28] Li W. , Li E. , and Wang S. , et al.Comparative Effects of Four Feed Attractants on Growth, Appetite, Digestion and Absorption in Juvenile Chinese Mitten Crab (*Eriocheir sinensis*), Aquaculture. (2025) 594, 10.1016/j.aquaculture.2024.741441, 741441.

[29] AOAC , Official Methods of Analysis of AOAC International, 1995, AOAC, Arlington, Va..

[30] Gweon H. S. , Bowes M. J. , and Moorhouse H. L. , et al.Contrasting Community Assembly Processes Structure Lotic Bacteria Metacommunities Along the River Continuum, Environmental Microbiology. (2021) 23, no. 1, 484–498, 10.1111/1462-2920.15337. 33258525 PMC7898806

[31] Robbins K. R. , Saxton A. M. , and Southern L. L. , Estimation of Nutrient Requirements Using Broken-Line Regression Analysis1, Journal of Animal Science. (2006) 84, no. suppl_13, E155–E165, 10.2527/2006.8413_supplE155x, 2-s2.0-33845635734. 16582088

[32] Liang X. , Han J. , and Xue M. , et al.Growth and Feed Intake Regulation Responses to Anorexia, Adaptation and Fasting in Japanese Seabss, Lateolabrax Japonicas When Fishmeal Is Totally Replaced by Plant Protein, Aquaculture. (2019) 498, 528–538, 10.1016/j.aquaculture.2018.09.010, 2-s2.0-85053078466.

[33] Pan M. V. , Cadiz R. E. , Mameloco E. J. G. , and Traifalgar R. F. M. , Squid Industry By-Product Hydrolysate Supplementation Enhances Growth Performance of *penaeus monodon* Fed Plant Protein-Based Diets Without Fish Meal, Frontiers in Sustainable Food Systems. (2022) 6, 10.3389/fsufs.2022.1027753, 1027753.

[34] Biswas P. , Patel A. B. , and Saha H. , Effect of Dietary Incorporation of Chemo-Attractants on Growth and Survival During Seed Rearing of Ompok Bimaculatus (bloch), Turkish Journal of Fisheries and Aquatic Sciences. (2018) 18, 491–499.

[35] Mian S. , Saha S. , and Rabbani G. , et al.Dietary Inosine Monophosphate Improved Growth, Feed Utilization, Blood Biochemical Characteristics, and Intestinal Histo-Morphology of Slow Growing Golden Mahseer (*Tor putitora*), Animal Feed Science and Technology. (2023) 295, 10.1016/j.anifeedsci.2022.115545, 115545.

[36] Taklu M. , Islami H. R. , Mousavi S. A. , and Jourdehi A. Y. , Nucleotide Supplementation in the Diet of Sterlet Sturgeon (*Acipenser ruthenus*): Improved Zootechnical Performance, Biochemical Indices, and Immune Responses, Animal Feed Science and Technology. (2022) 288, 10.1016/j.anifeedsci.2022.115322, 115322.

[37] Vogt G. , Synthesis of Digestive Enzymes, Food Processing, and Nutrient Absorption in Decapod Crustaceans: A Comparison to the Mammalian Model of Digestion, Zoology. (2021) 147, 10.1016/j.zool.2021.125945, 125945. 34217027

[38] Garvey T. Q.III, Hyman P. E. , and Isselbacher K. J. , *γ*-Glutamyl Transpeptidase of Rat Intestine: Localization and Possible Role in Amino Acid Transport, Gastroenterology. (1976) 71, no. 5, 778–785, 10.1016/S0016-5085(76)80360-5, 2-s2.0-0017151158. 9332

[39] Inoue M. , Glutathionists in the Battlefield of Gamma-Glutamyl Cycle, Archives of Biochemistry and Biophysics. (2016) 595, 61–63, 10.1016/j.abb.2015.11.023, 2-s2.0-84968901866. 27095217

[40] Fuentes J. , Fonseca F. , and Gregório S. , et al.High Plant Protein Diet Impairs Growth Performance and Intestinal Integrity in Greater Amberjack (*Seriola dumerili*): Molecular and Physiological Insights, Aquaculture. (2025) 597, 10.1016/j.aquaculture.2024.741925, 741925.

[41] Willora F. P. , Vatsos I. N. , and Mallioris P. , et al.Replacement of Fishmeal With Plant Protein in the Diets of Juvenile Lumpfish (*Cyclopterus lumpus*, L. 1758): Effects on Digestive Enzymes and Microscopic Structure of the Digestive Tract, Aquaculture. (2022) 561, 10.1016/j.aquaculture.2022.738601, 738601.

[42] Segarra S. , Chau T. , Hoang P. , and Tran L. , Immunoregulation and Resistance to Aquatic Pathogens With Dietary Nucleotides in Pacific White Shrimp, *Litopenaeus vannamei* , Fishes. (2023) 8, no. 6, 10.3390/fishes8060308, 308.

[43] Shiau S.-Y. , Gabaudan J. , and Lin Y.-H. , Dietary Nucleotide Supplementation Enhances Immune Responses and Survival to Streptococcus Iniae in Hybrid Tilapia Fed Diet Containing Low Fish Meal, Aquaculture Reports. (2015) 2, 77–81, 10.1016/j.aqrep.2015.08.002, 2-s2.0-84940513998.

[44] Tahmasebi-Kohyani A. , Keyvanshokooh S. , Nematollahi A. , Mahmoudi N. , and Pasha-Zanoosi H. , Dietary Administration of Nucleotides to Enhance Growth, Humoral Immune Responses, and Disease Resistance of the Rainbow Trout (*Oncorhynchus mykiss*) Fingerlings, Fish & Shellfish Immunology. (2011) 30, no. 1, 189–193, 10.1016/j.fsi.2010.10.005, 2-s2.0-78650709084. 20955799

[45] Han H. , He N. , and Pan E. , et al.Disruption of the Intestinal Barrier by Avermectin in Carp Involves Oxidative Stress and Apoptosis and Leads to Intestinal Inflammation, Pesticide Biochemistry and Physiology. (2023) 195, 10.1016/j.pestbp.2023.105531, 105531. 37666586

[46] Sørensen S. L. , Park Y. , and Gong Y. , et al.Nutrient Digestibility, Growth, Mucosal Barrier Status, and Activity of Leucocytes From Head Kidney of Atlantic Salmon Fed Marine- or Plant-Derived Protein and Lipid Sources, Frontiers in Immunology. (2021) 11, 10.3389/fimmu.2020.623726, 623726. 33679713 PMC7934624

[47] Tie H.-M. , Feng L. , and Jiang W.-D. , et al.Dietary Exogenous Supplementation of Nucleotides Strengthens the Disease Resistance, Antioxidant Capacity and Immunity in the Gill of On-Growing Grass Carp (*Ctenopharyngodon idella*) Following a Challenge With *flavobacterium columnare* , Aquaculture. (2021) 540, 10.1016/j.aquaculture.2021.736729, 736729.

[48] Tie H.-M. , Wu P. , and Jiang W.-D. , et al.Dietary Nucleotides Supplementation Affect the Physicochemical Properties, Amino Acid and Fatty Acid Constituents, Apoptosis and Antioxidant Mechanisms in Grass Carp (*Ctenopharyngodon idellus*) Muscle, Aquaculture. (2019) 502, 312–325, 10.1016/j.aquaculture.2018.12.045, 2-s2.0-85058943577.

[49] Liu W. , Zhao C. , Wang P. , Wang S. , Lin H. , and Qiu L. , The Response of Glutathione Peroxidase 1 and Glutathione Peroxidase 7 Under Different Oxidative Stresses in Black Tiger Shrimp, *Penaeus monodon* , Comparative Biochemistry and Physiology Part B: Biochemistry and Molecular Biology. (2018) 217, 1–13, 10.1016/j.cbpb.2017.12.009, 2-s2.0-85038104450. 29246783

[50] Wang L. , Wu J. , Wang W.-N. , Cai D.-X. , Liu Y. , and Wang A.-L. , Glutathione Peroxidase From the White Shrimp *litopenaeus vannamei*: Characterization and Its Regulation Upon pH and Cd Exposure, Ecotoxicology. (2012) 21, no. 6, 1585–1592, 10.1007/s10646-012-0942-z, 2-s2.0-84864873399. 22684731

[51] Gibellini F. and Smith T. K. , The Kennedy Pathway—*De novo* Synthesis of Phosphatidylethanolamine and Phosphatidylcholine, IUBMB Life. (2010) 62, no. 6, 414–428, 10.1002/iub.337, 2-s2.0-77953591461. 20503434

[52] Pavlovic Z. and Bakovic M. , Regulation of Phosphatidylethanolamine Homeostasis—the Critical Role of CTP: Phosphoethanolamine Cytidylyltransferase (Pcyt2), International Journal of Molecular Sciences. (2013) 14, no. 2, 2529–2550, 10.3390/ijms14022529, 2-s2.0-84875114132. 23354482 PMC3588000

[53] Pan Y.-H. , Zhang Y. , and Cui J. , et al.Adaptation of Phenylalanine and Tyrosine Catabolic Pathway to Hibernation in Bats, PLoS ONE. (2013) 8, no. 4, 10.1371/journal.pone.0062039, 2-s2.0-84876438556, e62039. 23620802 PMC3631164

[54] Huyben D. , Roehe B. K. , Bekaert M. , Ruyter B. , and Glencross B. , Dietary Lipid: Protein Ratio and n-3 Long-Chain Polyunsaturated Fatty Acids Alters the Gut Microbiome of Atlantic Salmon Under Hypoxic and Normoxic Conditions, Frontiers in Microbiology. (2020) 11, 10.3389/fmicb.2020.589898, 589898. 33424792 PMC7785582

[55] Bharti M. , Nagar S. , Khurana H. , and Negi R. K. , Metagenomic Insights to Understand the Role of Polluted River Yamuna in Shaping the Gut Microbial Communities of Two Invasive Fish Species, Archives of Microbiology. (2022) 204, no. 8, 10.1007/s00203-022-03127-x, 509. 35859219

[56] Jiang J. , Li W. , and Wu Y. , et al.Effects of Cadmium Exposure on Intestinal Microflora of Cipangopaludina Cathayensis, Frontiers in Microbiology. (2022) 13, 10.3389/fmicb.2022.984757. PMC9393624 36003941

[57] Bouchon D. , Zimmer M. , and Dittmer J. , The Terrestrial Isopod Microbiome: An All-in-One Toolbox for Animal–microbe Interactions of Ecological Relevance, Frontiers in Microbiology. (2016) 7, 10.3389/fmicb.2016.01472, 2-s2.0-84993971951, 1472. 27721806 PMC5033963

[58] Ni J. , Ren L. , Liang Y. , Ma Y. , and Xiong H. , Modulatory Effects of Selenium Nanoparticles on Gut Microbiota and Metabolites of Juvenile Nile Tilapia (*Oreochromis niloticus*) by Microbiome-Metabolomic Analysis, Aquaculture Reports. (2025) 40, 10.1016/j.aqrep.2025.102627, 102627.

[59] Martínez-García M. , Brazel D. M. , and Swan B. , et al.Capturing Single Cell Genomes of Active Polysaccharide Degraders: An Unexpected Contribution of Verrucomicrobia, PLoS ONE. (2012) 7, no. 4, 10.1371/journal.pone.0035314, 2-s2.0-84860008730, e35314. 22536372 PMC3335022

[60] Faust K. and Raes J. , Microbial Interactions: From Networks to Models, Nature Reviews Microbiology. (2012) 10, no. 8, 538–550, 10.1038/nrmicro2832, 2-s2.0-84863920287. 22796884

[61] Guo X. , Ran C. , Zhang Z. , He S. , Jin M. , and Zhou Z. , The Growth-Promoting Effect of Dietary Nucleotides in Fish Is Associated With an Intestinal Microbiota-Mediated Reduction in Energy Expenditure, The Journal of Nutrition. (2017) 147, no. 5, 781–788, 10.3945/jn.116.245506, 2-s2.0-85020193778. 28356434

[62] Cosgrove M. , Nucleotides, Nutrition. (1998) 14, no. 10, 748–751, 10.1016/S0899-9007(98)00075-6, 2-s2.0-0032188789. 9785353

