# Supplementary material for: Dietary Nucleotides Improve Growth of Juvenile Eriocheir sinensis Under a Low‐Fish Meal Diet via Enhanced Feed Intake and Gut Health
Source: Aquac Nutr. 2025 Dec 9;2025:8633112. doi: 10.1155/anu/8633112 (PMC12714124; doi:10.1155/anu/8633112)
Supplement: Supplementary file 1 — Supporting Information 1 Table S1. Nucleotide composition of experimental diets (mg/100g dry matter). Table S2. Primer pair sequences of the genes used for real‐time PCR (qPCR). Table S3. Summary of principal component analysis of host physiological indicators. Table S4. Significance of marginal effects of host physiological principal components on gut microbiota structure in redundancy analysis. Figure S1. Effects of dietary nucleotide supplementation on whole‐body crude lipid and crude ash of juvenile Eriocheir sinensis fed low‐fish meal diets. Figure S2 Identification and phylum distribution of key bacterial species characterizing the gut microbiota of juvenile E. sinensis across different dietary treatments. [file ANU-2025-8633112-s002.docx]

**Table S1** Nucleotide composition of experimental diets (mg/100g dry matter).

| **Nucleotide**  **content** | **35%fishmeal**  **Con** | **15% fishmeal supplemented with nucleotides (g/kg diet)** | | | | | | **Detection** |
| --- | --- | --- | --- | --- | --- | --- | --- | --- |
|  |  | **0** | **0.3** | **0.6** | **0.9** | **1.2** | **2.4** |  |
| CMP | ND* | ND | ND | 12.60 | 14.30 | 20.30 | 44.40 | 3.30 |
| AMP | 16.06 | 9.29 | 13.90 | 20.04 | 24.38 | 29.91 | 53.47 | 5.00 |
| UMP | 104.06 | 50.27 | 55.80 | 61.64 | 71.63 | 78.54 | 107.12 | 5.00 |
| GMP | 4.64 | 4.12 | 8.60 | 13.54 | 18.68 | 23.89 | 45.20 | 5.00 |
| IMP | 103.55 | 43.21 | 47.17 | 51.57 | 57.52 | 61.25 | 80.28 | 6.70 |
| Total | 228.32 | 106.88 | 125.47 | 159.39 | 186.51 | 213.89 | 330.47 | / |

*ND = Not detected (below detection limit)

**Table S2** Primer pair sequences of the genes used for real-time PCR (qPCR).

| **Primers** | **Position** | **Primer sequence** | **Reference** |
| --- | --- | --- | --- |
| *NPYR* | Forward | CCACCAAGATGTCCGC | (Zhang et al., 2022) |
|  | Reverse | GAACAGGTCCCCGATG |  |
| *GABA2* | Forward | CCAATGTTATGCGAGGTGGC | XM050878665.1 |
|  | Reverse | TGGCCCCGTATATCCAGACA |  |
| *Ghrelin* | Forward | CTTCCTCAGCCCTTCACAAA | (Wang et al., 2024) |
|  | Reverse | AGACGCTGAATGATCTCCTG |  |
| *LEPR* | Forward | TCGAGGTGCCACAAAAGTCT | XM050866897.1 |
|  | Reverse | GGGTCACCAGTGCTTTCAGA |  |
| *CCKR* | Forward | CCTCGTGAATCTGGCG | (Zhang et al., 2022) |
|  | Reverse | CGTATGGCTCTGTCGG |  |
| *TOR* | Forward | GCAGCCCCAAGGAGATGAAA | (Zhang et al., 2022) |
|  | Reverse | ACAGTCGAAACGCCCTCATC |  |
| *AKT* | Forward | GGCAAAGTTATCCTCTGCCG | (Zhang et al., 2022) |
|  | Reverse | ATCGACTACTTGCAGGACCC |  |
| *4E-BP* | Forward | CACGAAACCGACTACTGC | (Zhang et al., 2022) |
|  | Reverse | CCAAGACCTGATGATGAAC |  |
| *p38-MAPK* | Forward | CACTCATGGGTGCTGACCTC | (Cheng et al., 2024) |
|  | Reverse | TACTTGAGGCCTCGCAACAC |  |
| *EsRelish* | Forward | ACAACATGACGAGCGTGACT | GQ871279.1 |
|  | Reverse | TCCAGGACAGCAACACACTC |  |
| *Bax* | Forward | GTCAGTGAACCTCAGCTGCAT | XM_050860151.1 |
|  | Reverse | CACAGCCACATCACCCACGAA |  |
| *Bcl2* | Forward | CAGGAAGGAGGATGGACCAAAA | XM_050860189.1 |
|  | Reverse | ACCAAGACCAATGGCAGTCC |  |
| *S27* | Forward | GGTCGATGACAATGGCAAGA | (Zhang et al., 2022) |
|  | Reverse | CCACAGTACTGGCGGTCAAA |  |

**References**

Cheng Y, Li C, Chen Y, Liu H, Wan H, Kang X, et al. Water pollutant cadmium-induced intestinal inflammation in *Eriocheir sinensis* and potential therapeutic drugs. Sci Total Environ 2024;955:177132. https://doi.org/10.1016/j.scitotenv.2024.177132.

Wang A, Fu Y, Fu L, Li M, Xu J, Guo X, et al. Dietary monosodium glutamate affects the growth and feed utilization of *Eriocheir sinensis* by regulating the appetite related genes expression. Aquac Rep 2024;38:102310. https://doi.org/10.1016/j.aqrep.2024.102310.

Zhang C, Wang X, Su R, He J, Liu S, Huang Q, et al. Dietary gamma-aminobutyric acid (GABA) supplementation increases food intake, influences the expression of feeding-related genes and improves digestion and growth of Chinese mitten crab (*Eriocheir sinensis*). Aquaculture 2022;546:737332. https://doi.org/10.1016/j.aquaculture.2021.737332.

**Table S3** Summary of Principal Component Analysis of host physiological indicators.

|  | Eigenvalue | Variance (%) | Cumulative Variance (%) |
| --- | --- | --- | --- |
| PC1 | 9.02 | 23.13 | 23.13 |
| PC2 | 6.84 | 17.55 | 40.68 |
| PC3 | 4.1 | 10.52 | 51.19 |
| PC4 | 3.78 | 9.7 | 60.89 |
| PC5 | 3.38 | 8.66 | 69.55 |
| PC6 | 2.29 | 5.88 | 75.42 |
| PC7 | 2.14 | 5.48 | 80.9 |

**Table S4** Significance of marginal effects of host physiological principal components on gut microbiota structure in Redundancy Analysis ¹.

| Principal Component | Variance Explained | F Value | P Value |
| --- | --- | --- | --- |
| PC1 | 1.996 | 2.029 | 0.007** |
| PC2 | 0.900 | 0.914 | 0.583 |
| PC3 | 0.837 | 0.851 | 0.720 |
| PC4 | 0.808 | 0.821 | 0.779 |
| PC5 | 1.121 | 1.139 | 0.245 |
| PC6 | 0.922 | 0.937 | 0.548 |
| PC7 | 0.782 | 0.795 | 0.839 |

¹ Results derived from permutation tests (999 permutations) assessing the unique contribution of each of the seven host PCs (derived from physiological indicators) in explaining the variance of Hellinger-transformed gut microbiota OTU data. ** *p* < 0.01.


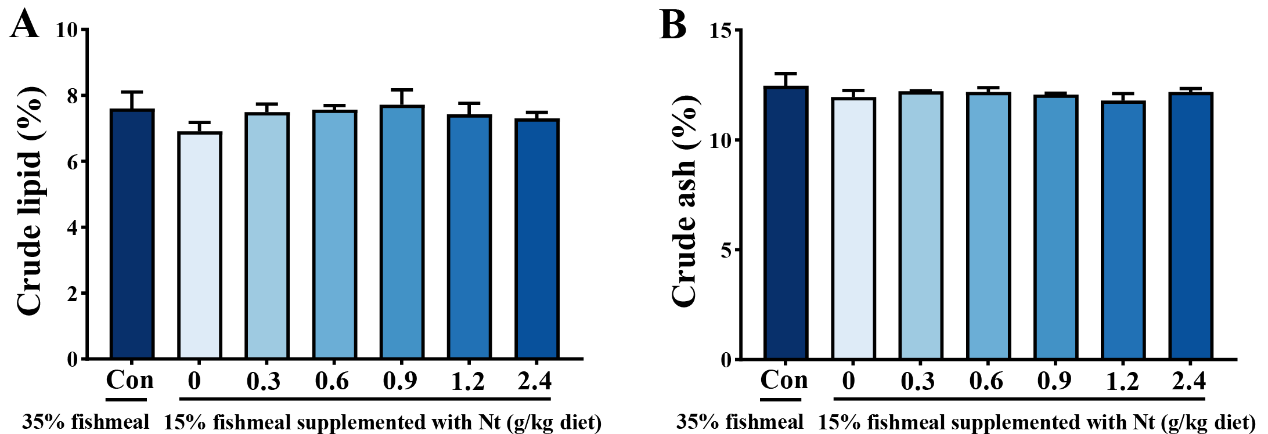
**Fig S1** Effects of dietary nucleotide supplementation on whole-body crude lipid and crude ash of juvenile *E. sinensis* fed low fishmeal diets.


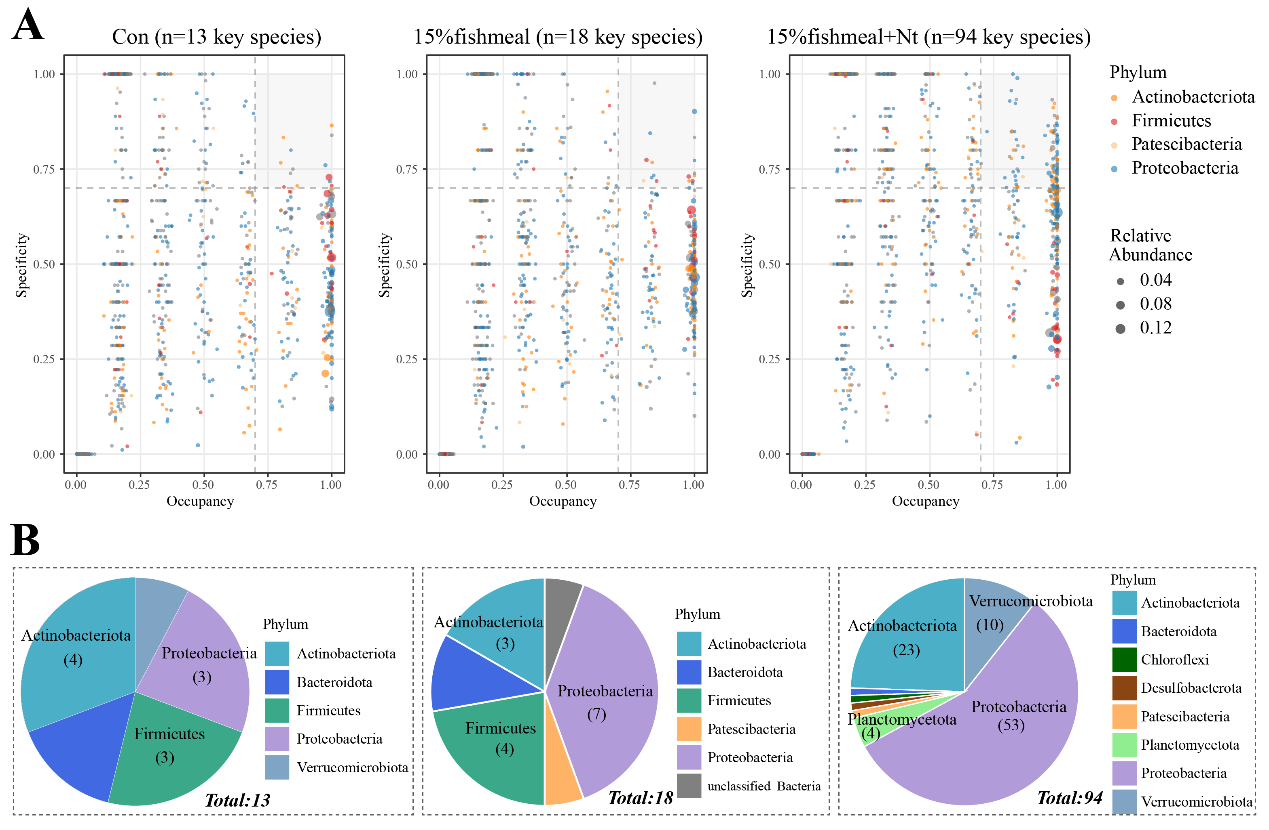


**Fig S2** Identification and phylum distribution of key bacterial species characterizing the gut microbiota of juvenile *E. sinensis* across different dietary treatments¹. (A) Specificity-Occupancy plots identifying key bacterial species strongly associated with each treatment group. Specificity reflects the average relative abundance within the group, while Occupancy reflects the frequency across samples within the group. Point color indicates Phylum and size indicates Relative Abundance. The total number of key species identified per group is noted. (B) Phylum-level composition of the key species identified in (A) for each respective group. Numbers indicate the count of key species belonging to each phylum.

¹ Dietary treatments: Control (Con, 35% fishmeal), 15% fishmeal (0 g/kg nucleotide), and 15% fishmeal + Nucleotides (0.9 g/kg nucleotide).
