# Supplementary material for: Dietary Nucleotides Improve Growth of Juvenile Eriocheir sinensis Under a Low‐Fish Meal Diet via Enhanced Feed Intake and Gut Health
Source: Aquac Nutr. 2025 Dec 9;2025:8633112. doi: 10.1155/anu/8633112 (PMC12714124; doi:10.1155/anu/8633112)
Supplement: Supplementary file 2 — Supporting Information 2 File S2. ARRIVE 2.0 Author Checklist completed for this study. [file ANU-2025-8633112-s001.docx]

**Supplementary Description : The ARRIVE guidelines 2.0: author checklist**

| Item | Recommendation | Section/Line Number, or Reason for Not Reporting |
| --- | --- | --- |
| Study design | 1 For each experiment, provide brief details of study design including: |  |
|  | 1. The groups being compared, including control groups. If no control group has been used, the rationale should be stated. | Line 117-119 |
|  | 1. The experimental unit (e.g., a single animal, litter, or cage of animals). | Line 125-127 |
| Sample size | 2 a. Specify the exact number of experimental units allocated to each group, and the total number in each experiment. Also indicate the total number of animals used. | Line 125-127; Line 200-201; Line 214 |
|  | 1. Explain how the sample size was decided. Provide details of any a priori sample size calculation, if done. | Line 125 |
| Inclusion and exclusion criteria | 3 a. Describe any criteria used for including and excluding animals (or experimental units) during the experiment, and data points during the analysis. Specify if these criteria were established a priori. If no criteria were set, state this explicitly. | Section 2.2 |
|  | 1. For each experimental group, report any animals, experimental units, or data points not included in the analysis and explain why. If there were no exclusions, state so. | Line 144-146 |
|  | 1. For each analysis, report the exact value of n in each experimental group. | Section figure captions |
| Randomisation | 4 a. State whether randomisation was used to allocate experimental units to control and treatment groups. If done, provide the method used to generate the randomisation sequence. | Line 126 |
|  | 1. Describe the strategy used to minimise potential confounders such as the order of treatments and measurements, or animal/cage location. If confounders were not controlled, state this explicitly. | Line 127-131 |
| Blinding | 5 Describe who was aware of the group allocation at the different stages of the experiment (during the allocation, the conduct of the experiment, the outcome assessment, and the data analysis). | Husbandry staff could not be blinded during the rearing period because the test diets differed in formulation and pellet characteristics |
| Outcome measures | 6 a. Clearly define all outcome measures assessed (e.g., cell death, molecular markers, or behavioural changes). | Line 141-144；  Section 2.5 |
|  | 1. For hypothesis-testing studies, specify the primary outcome measure, i.e., the outcome measure that was used to determine the sample size. | Line 234. Before data collection we pre-specified weight gain (WG, %) as the primary outcome for hypothesis testing. Specific growth rate (SGR) and 1-h feed intake (1h FI) were designated as secondary outcomes supporting interpretation. |
| Statistical methods | 7 a. Provide details of the statistical methods used for each analysis, including software used. | Section 2.5 |
|  | 1. Describe any methods used to assess whether the data met the assumptions of the statistical approach, and what was done if the assumptions were not met. | Section 2.5 |
| Experimental animals | 8 a. Provide species-appropriate details of the animals used, including species, strain and substrain, sex, age or developmental stage, and, if relevant, weight. | Line 123-125 |
|  | 1. Provide further relevant information on the provenance of animals, health/immune status, genetic modification status, genotype, and any previous procedures. | Line 121-123 |
| Experimental procedures | 9 For each experimental group, including controls, describe the procedures in enough detail to allow others to replicate them, including: |  |
|  | 1. What was done, how it was done, and what was used. | Section 2.1 |
|  | 1. When and how often. | Line 133-135 |
|  | 1. Where (including detail of any acclimatisation periods). | Line 123-125 |
|  | 1. Why (provide rationale for procedures). | Section 2.4 |
| Results | 10 For each experiment conducted, including independent replications, report:   1. Summary/descriptive statistics for each experimental group, with a measure of variability where applicable (e.g., mean and SD, or median and range). | Section figure captions, “Data are presented as means ± SEM (n = 5).” |
|  | 1. If applicable, the effect size with a confidence interval. | Effect sizes and 95% confidence intervals were not calculated or reported in the current manuscript; results are presented as mean ± SEM with p-values. |
